# MIRO1 mutation leads to metabolic maladaptation resulting in Parkinson’s disease-associated dopaminergic neuron loss

**DOI:** 10.1038/s41540-025-00509-x

**Published:** 2025-04-17

**Authors:** Alise Zagare, Thomas Sauter, Kyriaki Barmpa, Maria Pacheco, Rejko Krüger, Jens Christian Schwamborn, Claudia Saraiva

**Affiliations:** 1 Developmental and Cellular Biology, Luxembourg Centre for Systems Biomedicine (LCSB), University of Luxembourg, 2, place de l’Université, L-4365 Esch-sur-Alzette, Luxembourg; 2 Systems Biology and Epigenetics Group, Department of Life Sciences and Medicine, University of Luxembourg, 2, place de l’Université, L-4365 Esch-sur-Alzette, Luxembourg; 3 Translational Neuroscience, Luxembourg Centre for Systems Biomedicine (LCSB), University of Luxembourg, 2, place de l’Université, L-4365 Esch-sur-Alzette, Luxembourg; 4 Transversal Translational Medicine, Luxembourg Institute of Health (LIH), 1 A-B rue Thomas Edison, L-1445 Strassen, Luxembourg; 5 Parkinson Research Clinic, Centre Hospitalier de Luxembourg, 4, rue Ernest Barblé, L-1210 Luxembourg, Luxembourg

**Keywords:** Neuroscience, Stem cells, Multicellular systems, Bioenergetics

## Abstract

MIRO1 is a mitochondrial outer membrane protein important for mitochondrial distribution, dynamics and bioenergetics. Over the last decade, evidence has pointed to a link between MIRO1 and Parkinson’s disease (PD) pathogenesis. Moreover, a heterozygous MIRO1 mutation (p.R272Q) was identified in a PD patient, from which an iPSC-derived midbrain organoid model was derived, showing MIRO1 mutant-dependent selective loss of dopaminergic neurons. Herein, we use patient-specific iPSC-derived midbrain organoids carrying the MIRO1 p.R272Q mutation to further explore the cellular and molecular mechanisms involved in dopaminergic neuron degeneration. Using single-cell RNA sequencing (scRNAseq) analysis and metabolic modeling we show that the MIRO1 p.R272Q mutation affects the dopaminergic neuron developmental path leading to metabolic deficits and disrupted neuron-astrocyte metabolic crosstalk, which might represent an important pathogenic mechanism leading to their loss.

## Introduction

Parkinson’s disease (PD) is a multifactorial age-associated disorder characterized by the selective degeneration of dopaminergic neurons residing in the *substantia nigra pars compacta* of the midbrain and by the presence of proteinaceous inclusions (Lewy bodies and Lewy neurites). Available treatments for PD are symptomatic and restricted to either a time window or a subset of individuals^1^. Being the second most prevalent neurodegenerative disorder and the fastest growing one, PD poses several challenges at the social, economic and healthcare level^2^, which further supports the need to understand the mechanisms causing cell demise.

Most PD cases are idiopathic, with only 5% to 15% being familial (genetic component). Nevertheless, even in sporadic cases, genome-wide association studies have shown increased importance of both common and rare genetic variants^3^. In fact, a recent study predicted that *RHOT1* variations were among the top 5 rare genetic variants with the highest functional impact on PD^4^. *RHOT1* encodes MIRO1, a conserved mitochondrial-related protein, constituted by two GTPase domains flanking two EF-hand Ca^2+^-binding domains and a C-terminal transmembrane domain able to bind the mitochondrial outer membrane^5^. MIRO1 is an important regulator of mitochondria dynamics, including morphology, transport, mitophagy and Ca^2+^ homeostasis^6^. Recently, we have not only identified PD patients carrying heterozygous mutations on MIRO1^7,8^, but we also demonstrated that the MIRO1 p.R272Q mutation, affecting the 1^st^ EF-hand Ca^2+^-binding domain, is sufficient to promote loss of tyrosine hydroxylase (TH)-positive dopaminergic neurons in aged knock-in mice and patient-specific iPSC-derived midbrain organoids^9^. The MIRO1 p.R272Q mutation seems to cause mitochondrial dysfunction in iPSC-derived dopaminergic neurons and midbrain organoids, including increased oxidative stress, lower mitochondrial membrane potential and oxidative phosphorylation-associated energy deficits^9,10^. Deregulation of mitochondrial function and increased oxidative damage are well-established pathogenic mechanisms of PD^11^.

In addition, increasing evidence suggests a possible neurodevelopmental component as one of the PD “multi-hit” hypothesis triggers^12^. Variations related to altered differentiation of midbrain dopaminergic neurons^13–16^ and/or metabolic changes at the subcellular, cellular and system levels^17–20^ could be at the core of dopaminergic neuron degeneration. Metabolic alterations are especially relevant considering not only the neurons and astroglia intricately linked metabolism but also dopaminergic neurons’ poor myelination and highly complex and extensive branching architecture, which underlies their increased energy requirements^21,22^. Accordingly, a bioenergetic maladaptation has been observed in neurodegenerative diseases, including PD^23^.

We hypothesize that the MIRO1 p.R272Q mutation affects cellular metabolic processes, making dopaminergic neurons more susceptible to degeneration. This is supported by the fact that mitochondria are key players in energy metabolism as well as by MIRO1 role in proper mitochondrial function. Therefore, we use midbrain organoids, which have diverse cellular composition, to analyze dopaminergic neuron and astrocyte amounts at days 20, 30, 60, and 90 of culture. Then, by integrating single-cell transcriptomic data into the genome-scale metabolic model Recon3D, we further predict midbrain organoid metabolism at the single-cell level^24^ at days 30 and 60 of culture. This strategy allows us to gain some understanding of the complex metabolic interaction between neurons and astrocytes and the identification of metabolic disturbances associated with dopaminergic neuron loss.

## Results

### PD-MIRO1 mutant organoids show sustained dopaminergic neuron loss and altered astrocyte differentiation

**Fig. 1:**
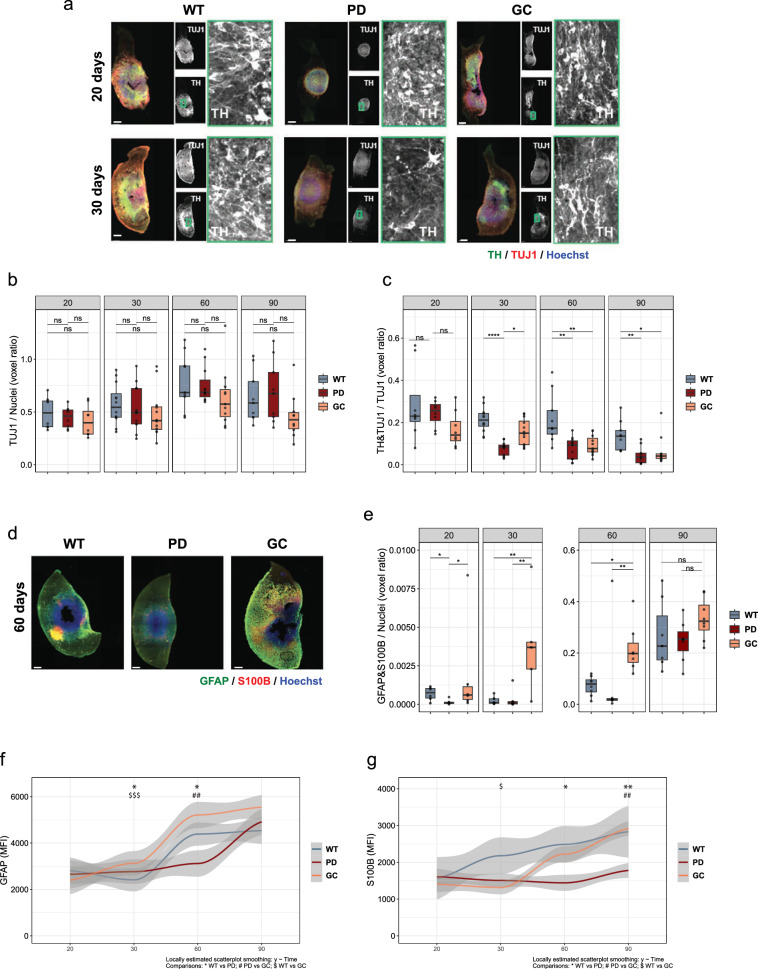
PD-MIRO1 mutant organoids show sustained dopaminergic neuron loss and altered astrocyte differentiation. **a** Representative images of 20 and 30 day old organoids staining for neurons (TUJ1, red), dopaminergic neurons (TH, green) and nuclei (Hoechst, blue). Scale bar = 200 µm. **b** Quantification of total neuronal population within organoids over time quantified by pixel volume ratio (TUJ1/nuclei). *n* = 8 to 12 from at least 4 independent organoid derivations. **c** Quantification of total dopaminergic neuron population within organoids over time quantified by pixel volume ratio ((TH + TUJ1)/TUJ1). **d** Representative Images of 60 day old astrocytes within WT and PD organoids using GFAP (green), S100B (red) and Hoechst (nuclei, blue). Scale bar = 200 µm. **e** Quantification of total astrocyte population within organoids over time quantified by pixel volume ratio ((GFAP + S100B)/nuclei). *n* = 6 to 8 from at least 3 independent derivations. **f** Expression levels of GFAP protein over time quantified by the mean intense fluorescence. Statistical significance was tested with Kruskall-Walis test.*p < 0.05 (WT vs PD); ^##^*p* < 0.01 (PD vs GC); ^$$$^*p* < 0.001 (WT vs GC). **g** Expression levels of GFAP protein over time quantified by the mean intense fluorescence. Statistical significance was tested with Kruskall-Walis test.*p < 0.05 (WT vs PD); ^$^p < 0.05 (WT vs GC); **p < 0.01 (WT vs PD); ^##^p < 0.01 (PD vs GC). **b**, **c**, **e** Statistical significance was tested with the Kruskall-Walis test and represented with ns *p* > 0.05; *p < 0.05; **p < 0.01; ***p < 0.001; ****p < 0.0001.

Midbrain organoids are multi-cellular complex structures resembling the human embryonic midbrain, which are capable of recapitulating key pathological features of genetic PD^15,16,25^. Recently, we showed that the MIRO1 p.R272Q mutation promotes the loss of dopaminergic neurons at day 30 of midbrain organoids culture, a phenotype accompanied by evidence of compromised mitochondrial function^9^. Nevertheless, the influence of the MIRO1 p.R272Q pathogenic mutation in midbrain organoids over time, both at the cellular and subcellular levels is not understood. Here, we generated midbrain organoids using an iPSC line from a PD patient carrying the heterozygous MIRO1 p.R272Q mutation (PD or PD-MIRO1), the respective isogenic control iPSC line (correction of the MIRO1 point mutation; GC), and an iPSC line from a sex- and age-matched healthy individual (WT) (Table 1). Cellular phenotypes related to the general neuronal population, dopaminergic neurons and astrocytes were then evaluated in the organoids at four time points: 20, 30, 60 and 90 days of culture (Fig. 1).

**Table 1:** Cell lines used to generate midbrain organoids

ID	Diagnosis	Genotype	Sex	Age of sampling	Age of onset	Reference/Source
**WT**	Healthy (Control)	wt/wt	Female	68		^17^
**PD**	PD	MIRO1 p.R272Q/wt	Female	78	70	^76^
**GC**	PD (background)	wt/wt	Female	78	70	^76^
	WT (genotype)					

The general neuronal population within organoids was evaluated using the class III beta-tubulin marker (TUJ1). The number of neurons observed in the three conditions was similar between all the time points analyzed (Fig. 1a, b). Next, the levels of the TH enzyme were investigated. TH is the rate-limiting enzyme in catecholamine biosynthesis, namely dopamine, and a classical marker for dopaminergic neurons. As expected, PD-MIRO1 mutant organoids have significantly less dopaminergic neurons compared to WT (Fig. 1a, c). The loss of TH-positive dopaminergic neurons was initially observed at day 30 of organoid culture and remained consistent in all the subsequent time points assessed. Of note, this phenotype is not detectable at day 20, suggesting that loss of dopaminergic neurons occurs during prolonged culturing. This is in accordance with what we had previously reported^9^. Interestingly, the GC control behaved similarly to the WT control, but only up to day 30 of midbrain organoid culture, after which it phenotypically resembled the PD organoids. This indicates that indeed the MIRO1 p.R272Q mutation has an important role in PD pathogenesis. However, it also suggests that, in this specific case, the genetic background of the individual does affect phenotypes, a phenomenon seen in other PD-related models, such as LRRK2-G2019S patient-specific iPSC-derived models^26,27^.

Next, we investigate the influence of the MIRO1 p.R272Q mutation on astrocytes. Astrocytes represent the major glial population in the adult human brain and play a critical role in the central nervous system’s proper function. Astrocytes ensure optimal neuronal function by providing metabolic support to neurons, participating in synaptic transmission and plasticity as well as in anti-oxidative and immune responses^28^. In midbrain organoids, mature astrocytes, characterized by double expression of astrocytic markers GFAP and S100B (Fig. 1d), are normally increasing over time, with a larger population being observed at time points later than day 30 of culture (Fig. 1e)^29^. Despite no significant alterations in the number of mature astrocytes (GFAP&S100B double-positive cells) between PD and WT conditions from day 30 onwards (Fig. 1e), changes in GFAP and S100B protein levels were detected (Fig. 1f, g). As expected, in WT conditions, both GFAP and S100B mean fluorescent intensity signal was lower at days 20 and 30 of culture, increasing at days 60 and 90 (Fig. 1f, g), consistent with the appearance of mature astrocyte (Fig. 1e). In the MIRO1 mutant organoids, GFAP mean fluorescent intensity was similar to WT at day 20, slightly (although significantly) higher at day 30, but significantly lower at day 60, and finally catching up to similar levels at day 90 (Fig. 1f). Similarly, PD organoids showed no differences in S100B mean fluorescent intensity values at days 20 and 30 compared with WT, however, significantly lower values were observed up to day 90 of organoid culture (Fig. 1g). These results suggest that, while the MIRO1 p.R272Q mutation does not affect the number of mature astrocytes, it may delay GFAP and S100B protein synthesis, which could potentially have an impact on the astrocyte function. The majority of the phenotypes described appeared to be MIRO1 p.R272Q mutation-specific, nevertheless, we did observe a contribution of the patient’s genetic background. Furthermore, the cellular trajectories over time and the absence of mitochondrial alterations in the GC case^9^ suggest an alternative mechanism by which TH loss is achieved. Therefore, to ensure relevant comparisons, subsequent experiments will focus on WT and MIRO1 mutant midbrain organoids.

Altogether, our results demonstrate a significant influence of MIRO1 p.R272Q mutation on the survival of TH-positive dopaminergic neurons in midbrain organoids, while also suggesting a potential, albeit less severe, effect on the astrocyte population.

### The altered differentiation path of dopaminergic neurons within PD-MIRO1 mutant organoids leads to tyrosine hydroxylase loss over time

**Fig. 2:**
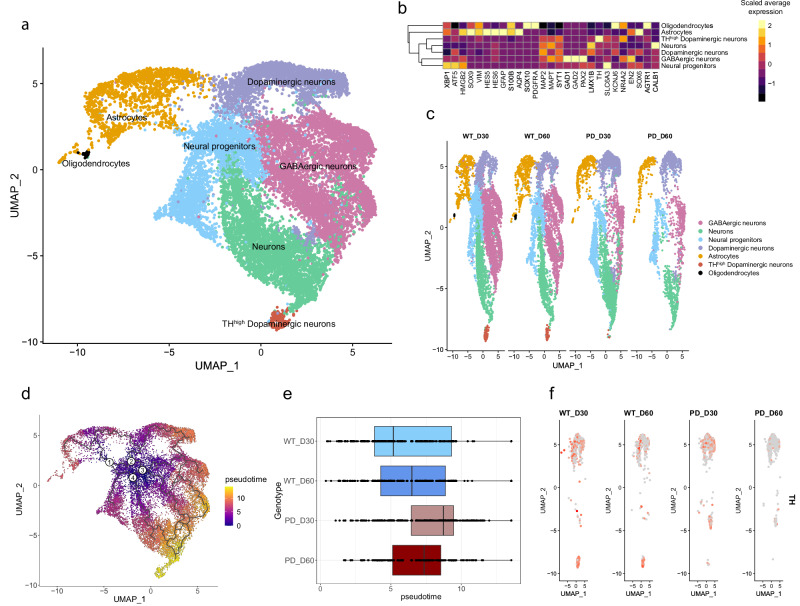
Altered differentiation path of dopaminergic neurons within PD-MIRO1 mutant organoids leads to their loss over time. **a** UMAP of Seurat object after integration of WT, PD and GC samples of day 30 and day 60 of differentiation. Dots represent single cells and colors represent cellular populations. **b** Heatmap representing cell type-specific marker expression. **c** Split UMAP plots of WT and PD samples of day 30 and day 60 of differentiation. **c** UMAP of pseudotime trajectory across cell population within the integrated Seurat object. **d** Pseudotime comparison between WT and PD dopaminergic neuron and TH^high^ dopaminergic neuron subset over time points. **f** Feature plot of TH expression in WT and PD dopaminergic neuron and TH^high^ dopaminergic neuron subset over time points.

To investigate the potential underlying causes of TH-positive dopaminergic neuron loss over time in the PD-MIRO1 case, we performed a single-cell transcriptomics analysis of midbrain organoids at 30 and 60 days of differentiation. We identified seven distinct cell clusters, including two different dopaminergic neuron clusters (Fig. 2a). Cellular identity was determined based on the cell type-specific marker expression (Fig. 2b)^9^. Neural progenitors demonstrated high expression of stemness and differentiation marker *HMGB2*. In addition, they showed high expression of *XBP1*, involved in the maturation of developing neurons, and *ATF5*, involved in glial differentiation, suggesting that neural progenitor cluster resembles neuroepithelial stem cells (NESCs) and captures their ability to give rise to neurons as well as glial cells. GABAergic neurons showed high expression of *GAD1* and *GAD2* both involved in the synthesis of GABA, and *PAX2* involved in GABAergic neuron development. Both dopaminergic neuron clusters were identified based on the expression of midbrain dopaminergic neuron identity markers *LMX1B*, *SOX6*, and *TH*. These markers are important for the specification of dopaminergic neurons as well as for the production of the enzyme responsible for the limiting step in the conversion of tyrosine into dopamine. Notably, one of the dopaminergic neuron clusters exhibited a strongly increased *TH* expression, hence was named TH^high^ dopaminergic neurons. These TH^high^ dopaminergic neurons also showed high expression of *NR4A2*, which is an essential transcription factor in the regulation of dopaminergic neuron differentiation. The high expression of *NR4A2*, as well as *SLC6A3*, *KCNJ6* and *AGTR1*, in the TH^high^ dopaminergic neuron cluster suggested its resemblance to A9 dopaminergic neurons, described as more vulnerable to degeneration in PD^30^. The second dopaminergic neuron cluster demonstrated high expression of both A9 dopaminergic neuron subtype marker *KCNJ6* and A10 subtype marker *CALB1*, suggesting their mixed identity or less advanced maturity state. This was further supported by the expression of *EN2* and *SOX6* in this cluster, with both markers demonstrating expression levels similar to those observed in neural progenitors. Additionally, we identified a general neuron cluster that displayed a similar marker expression to the dopaminergic neuron clusters, however, expressing fewer cell type-specific markers, suggesting incomplete specification. The astrocyte and oligodendrocyte clusters showed the absence of neuronal markers such as *MAP2*, *MAPT* and *SYT1*, while demonstrating high expression of astro glia markers — *SOX9*, *VIM*, *HES5*, *GFAP*, *S100B* — and oligodendrocyte markers like *SOX10* and *PDGFRA*. The high and simultaneous expression of astrocyte progenitor markers (*HMGB2*, *HES5* and *HES6*) as well as differentiated astrocyte markers (*GFAP* and *S100B*) in the astrocyte cluster indicates that this cluster comprises astrocytes at different maturity stages.

We observed that particularly, the TH^high^ dopaminergic neuron cluster showed a reduction in PD midbrain organoids over time (Fig. 2c, Fig. S1a, b), confirming their identity as a dopaminergic neuron cluster more vulnerable to degeneration. Further, we performed pseudotime trajectory analysis selecting as root nodes the branchpoints present in neural progenitors, assuming that they give rise to all other cell types in the midbrain organoids (Fig. 2d). We observed that TH^high^ dopaminergic neuron cluster demonstrated the highest pseudotime value, indicating their advanced state of maturity compared to other cell types (Fig. 2d, Fig. S2a, b). Furthermore, we observed that PD midbrain organoids at day 30 displayed an accelerated dopaminergic neuron differentiation state (analysis based on both dopaminergic neuron clusters), while further development was hampered over time (Fig. 2e). In contrast, WT midbrain organoids demonstrated gradual dopaminergic neuron differentiation over time, suggesting more precise regulation of developmental processes that might promote long-term sustainability of dopaminergic neurons in the WT case. Along with the loss of TH^high^ dopaminergic neuron cluster, PD midbrain organoids also exhibited a reduction of TH expression in the remaining dopaminergic neurons, which becomes more notorious by day 60 (Fig. 2f, Fig. S1a, b).

### MIRO1 mutant dopaminergic neurons endure mitochondrial respiratory dysfunction

**Fig. 3:**
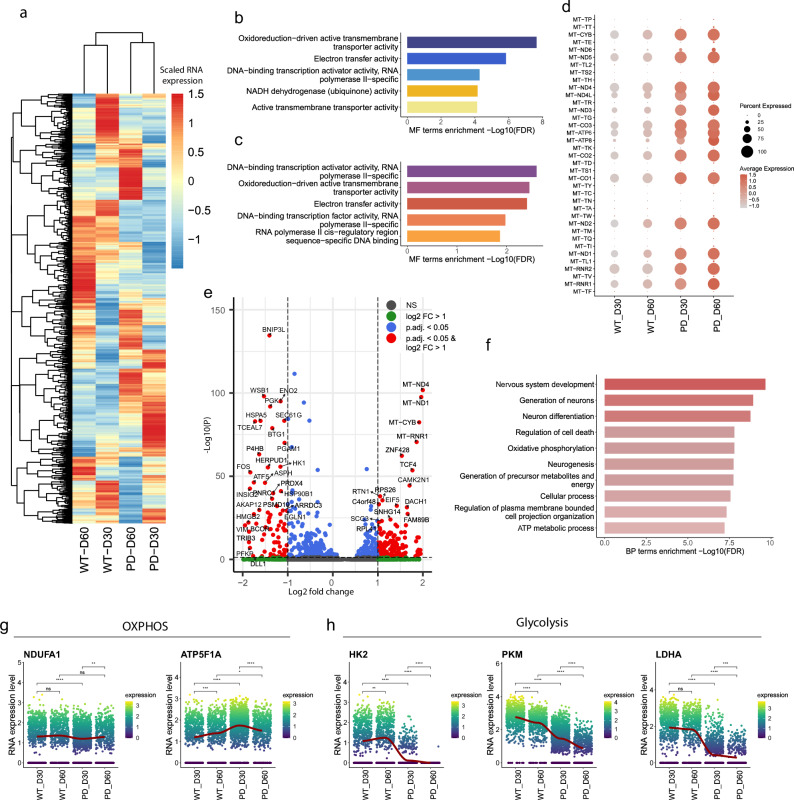
PD-MIRO1 mutant dopaminergic neurons endure mitochondrial respiratory dysfunction. **a** Heatmap of genes with a significant expression change along the pseudotime between day 30 and day 60 time points of WT and PD dopaminergic neurons. **b** Top five significantly enriched molecular function (MF) terms of top 100 genes with a significant expression change along the pseudotime between day 30 and day 60 time points of WT dopaminergic neurons. Each color represents a different MF term. **c** Top five significantly enriched molecular function terms of top 100 genes with a significant expression change along the pseudotime between day 30 and day 60 time points of PD dopaminergic neurons. Each color represents a different MF term. **d** Scaled expression of mitochondrial DNA genes in all conditions. **e** Volcano plot of differentially expressed genes between WT and PD dopaminergic neurons at day 60. **f** Top 10 significantly enriched biological processes of significantly differentially expressed genes (p.adjust.<0.05) between WT and PD dopaminergic neurons at day 60. **g** RNA expression of OXPHOS involved mitochondrial complex I subunit *NDUFA1* and mitochondrial complex V subunit *ATP5F1A* genes across all conditions. Statistical significance was tested with the Wilcoxon rank sum test. Significance represented with ns *p* > 0.05; *p < 0.05; **p < 0.01; ***p < 0.001; ****p < 0.0001. **h** RNA expression of glycolysis involved hexokinase 2 (*HK2*), pyruvate kinase (*PKM*) and lactate dehydrogenase A (*LDHA*) genes across all conditions. Statistical significance was tested with the Wilcoxon rank sum test. Significance represented with ns *p* > 0.05; *p < 0.05; **p < 0.01; ***p < 0.001; ****p < 0.0001.

Next, we wanted to identify genes regulating the developmental process within dopaminergic neurons in WT and PD cases. We performed a spatial autocorrelation analysis^31^ on a subset of both dopaminergic neuron clusters to determine genes whose expression changes along the pseudotime trajectory. All identified genes between day 30 and day 60 for WT and PD with the q-value < 0.05 were combined in a single list of genes to analyze their expression pattern across all conditions (Fig. 3a). We observed that the expression signature of genes switching on and off between the two time points was highly distinct between WT and PD dopaminergic neurons. Further, we performed a gene enrichment analysis of the top 100 genes with significant expression changes between the two time points for the WT and PD samples to determine the molecular function of the genes responsible for the developmental path of dopaminergic neurons (Table S1 and S2). The top five significantly enriched molecular function terms in WT dopaminergic neurons were associated with oxidoreduction-driven transmembrane transporter activity, electron transfer activity, NADH dehydrogenase or mitochondrial complex I activity, active transmembrane transport activity, and DNA-binding transcription activity (Fig. 3b). Similarly, the top five significantly enriched molecular function terms in PD included oxidoreduction-driven transmembrane transporter activity and electron transfer activity. However, three out of five top molecular functions were associated with RNA-polymerase activity (Fig. 3c). These results showed that the molecular function of the top 100 genes with the highest change between the time points of both WT and PD midbrain organoids was strongly associated with electron transport and oxidoreduction-driven transmembrane transporter activity. This indicates the essential role of mitochondria in the dopaminergic neuron developmental path. Therefore, we further investigated the expression of mitochondrial genes to evaluate the functional state of mitochondria in WT and PD cases. We observed strongly increased expression levels of mitochondrial DNA genes at both time points of PD dopaminergic neurons compared to WT (Fig. 3d), suggesting enhanced mitochondrial metabolism in PD. To further evaluate the consequences of an altered developmental path and overactivation of mitochondrial metabolism in PD, we looked at the differentially expressed genes (DEGs) between WT and PD dopaminergic neurons at the latest time point — day 60. We found in total 2403 DEGs (p.value < 0.05) (Fig. 3e). We then performed gene functional enrichment analysis of the top DEGs with p.adjust. <0.05 to identify the most affected biological process in PD dopaminergic neurons at this time point. The top 10 significantly enriched terms included biological processes related to neuronal development and differentiation as well as energy generation-associated metabolic pathways such as oxidative phosphorylation, precursor metabolite generation and ATP metabolism (Fig. 3f). In addition, we evaluated the expression of key metabolic genes of oxidative phosphorylation (OXPHOS) and glycolysis to assess metabolic differences between WT and PD midbrain dopaminergic neurons (Fig. 3g, h). We saw that the subunit *NDUFA1* of the mitochondrial complex I is significantly downregulated between WT and PD dopaminergic neurons at day 30, while at day 60 the expression levels are similar. At the same time, ATP synthase subunit ATP5F1A showed significantly higher expression levels at both time points in PD dopaminergic neurons. However, in the WT case, *ATP5F1A* expression was increasing over time, while in PD the expression of *ATP5F1A* was reduced over time, suggesting less robust mitochondrial respiratory function in PD dopaminergic neurons. Additionally, we analyzed two key enzymes of glycolysis to evaluate whether PD dopaminergic neurons might use an alternative pathway for ATP generation to compensate for dysregulated mitochondrial metabolic function. We observed that glycolysis rate-limiting enzyme hexokinase 2 (*HK2*) and the final glycolysis enzyme generating pyruvate and ATP — pyruvate kinase (*PKM*) — were significantly downregulated at both time points of PD dopaminergic neurons, suggesting reduced reliance on glycolysis for energy generation. Moreover, the absence of *HK2* at day 60 of PD dopaminergic neurons indicates that glucose is not the primary energy source. Instead, other energy sources, such as fatty acids or lactate from astrocytes, might be preferentially used in MIRO1 mutant dopaminergic neurons. Finally, we observed significantly reduced expression levels of lactate dehydrogenase A (*LDHA*) in the PD dopaminergic neurons. LHDA is necessary for maintaining glycolysis and regenerating NAD+ form NADH, further suggesting altered glycolysis function and potentially reduced levels of NAD+ cofactor in PD case. Indeed, reduced NAD+ cofactor levels have been observed in 30 day old PD-MIRO1 mutant organoids and other patient-specific iPSC-based PD models^17^.

### Multi-cell population modeling predicts the metabolic activity of midbrain organoids

**Fig. 4:**
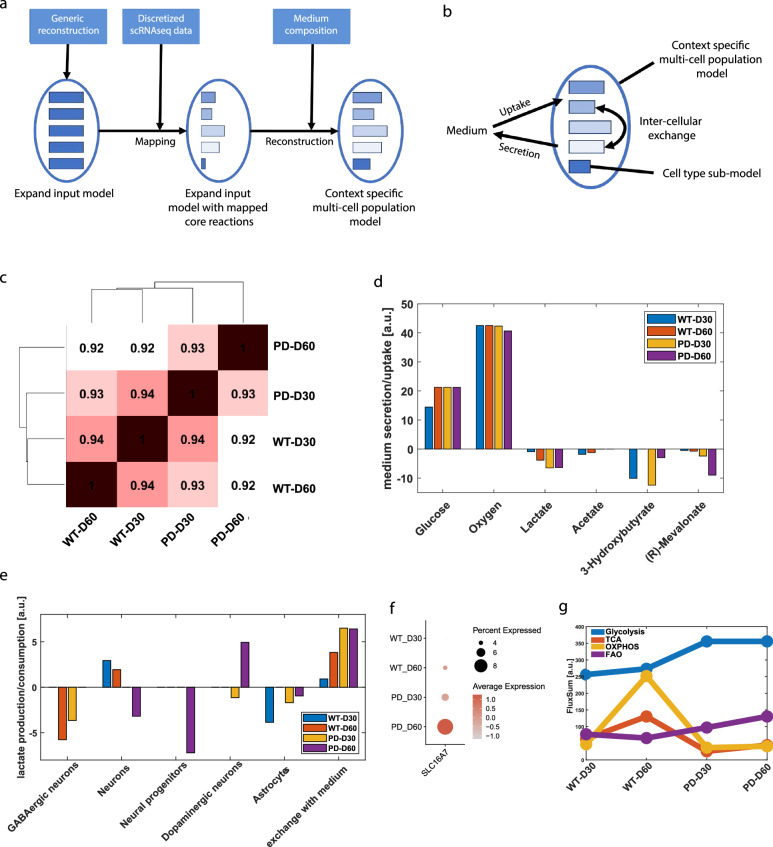
Multi-cell population modeling predicts metabolic activity of midbrain organoids. **a** Metabolic modeling workflow (scFASTCORMICS, see methods). **b** Schematic overview of reconstructed model (per one condition). **c** Model similarity. Jaccard similarity coefficient based on reaction presence calculated for each pair of multi-cell population models, ranging from 0 (dissimilar) to 1 (equal). **d** Predicted medium uptake and secretion rates of midbrain organoids for key metabolites. Positive values [a.u.] indicate uptake of the respective metabolite and negative values [a.u.] indicate secretion. **e** Predicted lactate inter-cellular exchange between different cell types. Negative values [a.u.] indicate production of the respective metabolite and positive values [a.u.] indicate uptake (or secretion to the medium). **f** Expression of *SLC16A7* in dopaminergic neurons of midbrain organoids. Dot size represents the cell percentage expressing the gene, colour represents the scaled average expression. **g** FluxSum estimates show the metabolic dependence of the midbrain organoid conditions in each energy pathway. Estimates are done by the sum of relevant metabolites (see Supplementary Table S5) within the respective pathway. TCA- tricarboxylic acid cycle; OXPHOS- oxidative phosphorylation; FAO- fatty acid oxidation.

To further elucidate the metabolic alterations linked to the MIRO1 p.R272Q mutation and explore dysregulated pathways in more detail, we used metabolic modeling. Metabolic network models were reconstructed with the scFASTCORMICS workflow^32^, by integrating the discretized scRNAseq data with the generic human metabolic reconstruction Recon3D and quantitative medium information (Fig. 4a, see Methods for details). The resulting multi-cell population model represents the metabolism of the complete organoid (context-specific multi-cell population model), as well as the metabolism of the different cell types (cell type sub-models). The sizes of the cell-type sub-models ranged from 3441 to 3646 in terms of the number of reactions (Table S3) and from 2441 to 2496 in terms of the number of metabolites (Table S4). The model allows to estimate the metabolic pathway activities of organoids in the different conditions, the respective medium uptake and secretion rates, as well as the putative metabolic crosstalk between the different cell types (inter-cellular exchange) (Fig. 4b). To get an overview of the metabolic alterations in midbrain organoids in the different conditions, we first assessed the similarity of reconstructed multi-cell population models considering the presence of biochemical reactions in the models. This showed a high similarity of 94% and 93% within the WT and PD conditions, respectively (Fig. 4c). The similarity between WT and PD conditions at day 30 was slightly higher (94%) than between WT and PD conditions at day 60 (92%). This difference may reflect the greater diversity and maturity of cells within the organoids or suggest more pronounced metabolic differences between PD and WT at later time points. Moreover, the strong similarity between the models also indicated the same metabolic reactions were present in WT and PD metabolic models. Therefore, we focused on assessing possible quantitative differences in the metabolic reaction fluxes.

**Fig. 5:**
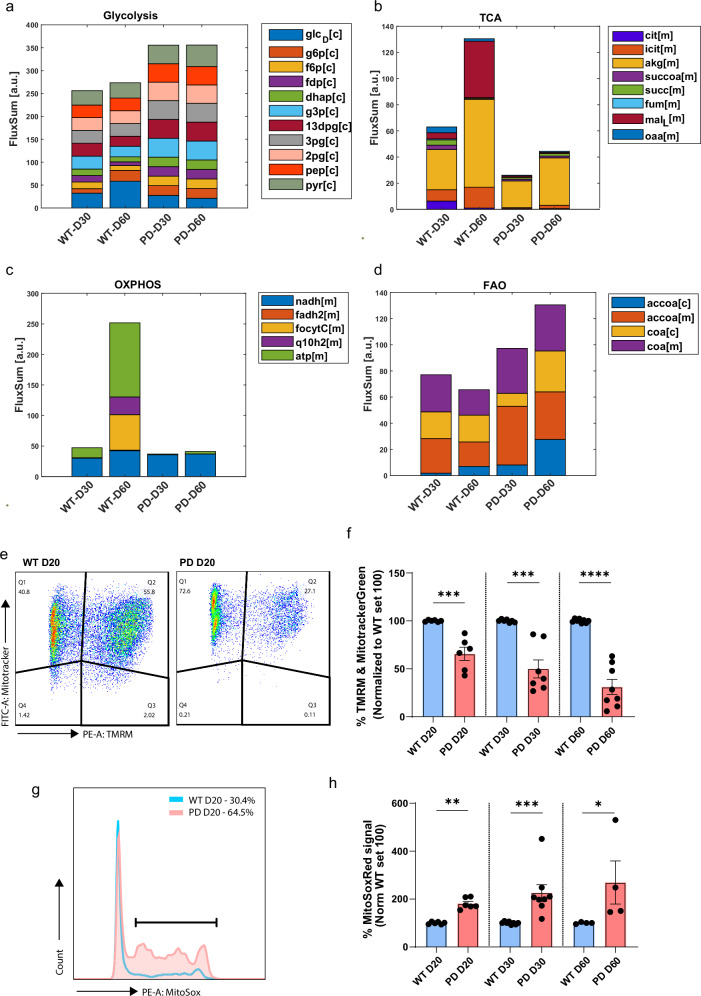
Multi-cell population modeling predicts differential activities of core metabolic pathways in WT vs PD conditions. **a**–**d** Metabolic activities are estimated by calculating the FluxSum (in [a.u.]) per metabolite across all cells of the organoid (sum of incoming metabolic fluxes in FBA solution) and are shown as bar plots of key metabolites per metabolic pathway (complete metabolite names corresponding to abbreviations are summarized at Supplementary Table S5). [c]-cytoplasm; [m]-mitochondria. **e** Dual Fluorescence scatter plots representing mitochondria (Q1&2) with intact membrane potential (Q2) in 20 day old organoids; WT (left) & PD (right). **f** The graph bar represents the percentage of mitochondria with intact membrane potential over time. *n* = 6 to 8 from at least 3 independent derivations. Statistical significance was tested with the Wilcoxon rank sum test. Significance represented with ***p < 0.001; ****p < 0.0001. **g** Representative histogram of MitoSox Red positive cells in 20 day old WT (blue) and PD (red) organoids. **h** The Graph bar represents the percentage of events with elevated mitochondrial ROS over time. *n* = 4 to 8 from at least 3 independent derivations. Statistical significance was tested with the Wilcoxon rank sum test. Significance represented with *p < 0.05; **p < 0.01; ***p < 0.001.

We performed a Flux Balance Analysis (FBA) with minimal cardinality of the resulting flux distribution (see methods) to predict the medium uptake and secretion rates, as well as inter-cellular exchange rates of all metabolites between cell types of the organoids in the different conditions. This analysis suggests major metabolic interactions between the cells, as different metabolites are produced by some cell types and consumed by others. Metabolites exchanging includes carbohydrates, amino acids, ketone bodies and prostaglandins, among others (File S1). Of note, the analysis was conducted using the default 2:1 O_2_/glucose ratio (https://cobrapy.readthedocs.io/en/latest/media.html), which seems to be representative of our experimental setting (organoid cultures) (Fig. 4, Fig. 5a–d). The physiological brain O_2_/glucose ratio of 5:1^33^ was also tested, with results showing minimal sensitivity to changes in maximal O_2_ uptake (Fig. S3). Of note, with these parameters we observed a minimal necessary oxygen uptake of 2.5-fold of the maximal glucose uptake.

Focusing in more detail on the major substrates glucose and oxygen, as well as the major glycolysis products, the FBA revealed similar glucose and oxygen uptake rates from the medium, but an increased secretion of lactate and other byproducts mostly in the PD condition (Fig. 4d, Fig. S3a). We observed that while in WT models lactate secretion increased from day 30 to day 60, in PD models, lactate secretion, was substantially higher at both time points. This is mainly due to lactate formation in neurons and reduced lactate secretion from astrocytes in PD (Fig. 4e, Fig. S3b). Generally, astrocytes are known to support neuronal energy needs by providing lactate as an energy substrate for neurons (lactate shuttle). However, we observed that particularly at day 60 the protein levels of GFAP and S100B in PD midbrain organoids were lower compared to WT midbrain organoids (Fig. 1f, g). This suggests that the already altered population of astrocytes in PD midbrain organoids might increase their metabolism in an attempt to compensate for dopaminergic neuron energy deficits. Considering the importance of the lactate shuttle, we looked at the lactate production and consumption rates between different cell types within the multi-cell metabolic models (Fig. 4e). Despite the low presence of fully mature astrocytes at day 30 (Fig. 1e), the FBA predictions demonstrated lactate shuttle from the astrocytes cluster (lactate production) to general neurons (lactate consumption) in the WT condition (Fig. 4e, in blue). However, we did not observe lactate uptake by WT dopaminergic neurons, suggesting that they do not rely on astrocyte-secreted lactate for metabolic support. Additionally, predictions indicated lactate production by GABAergic neurons (WT at day 60 and PD at day 30) and neural progenitors (PD at day 60), which might suggest high glycolytic activity of these cell types, also being a source of lactate. As observed in Fig. 4d, PD astrocytes demonstrated an increased lactate secretion rate, and specifically PD dopaminergic neurons at day 60 showed a high lactate uptake rate, which might indicate their increased need for metabolic support. This was supported by increased expression of neuronal lactate transporter Monocarboxylate transporter 2 (*MCT2*), also known as solute carrier family 16 member 7 (*SLC16A7*), particularly in PD dopaminergic neurons at day 60 (Fig. 4f). At the same time, PD dopaminergic neurons showed lactate production at day 30, suggesting their glycolytic activity and implying that pyruvate is rather secreted as lactate than used to fuel the mitochondrial tricarboxylic acid cycle (TCA). Overall, these results suggest the inability of PD dopaminergic neurons to generate enough energy to sustain its functions, highlighting the system reorganization, namely at the astrocytic function, to compensate, as long as possible, for these deficits.

Furthermore, FBA predicted an increased secretion rate of acetate in WT metabolic models compared to PD (Fig. 4d). Acetate can be produced from pyruvate to support acetyl-CoA pools and is known as an astrocyte-specific substrate, thus suggested as a marker for astrocyte metabolism^34,35^. The predicted elevated acetate secretion rate in WT models compared to PD might suggest that in the PD case all available acetate is used to fuel astrocyte metabolism rather than being secreted into the media. Additionally, FBA predicted an increased secretion of metabolite 3-hydroxybutyrate in PD at both time points compared to WT models (Fig. 4d). 3-hydroxybutyrate is a major ketone body known to be a primary energy source in the brain in case of low glucose^36^. 3-hydroxybutyrate is uptaken by brain cells, but not secreted. However, 3-hydroxybutyrate can be synthesized by astrocytes and has been shown to increase mitochondrial respiration, particularly in neurons^37^, further suggesting increased astrocyte support to neurons in the PD case. Furthermore, the FBA predicted alterations in the R-Mevalonate pathway. This pathway is known to produce cholesterol and other important intermediates crucial for neuronal functionality from acetyl-CoA. In addition, it regulates nutrient uptake and cell growth^38^. Altogether, these results demonstrate a strong effect of the MIRO1 mutation on cell metabolic regulation, particularly affecting neuron-astrocyte metabolic crosstalk.

To compare and summarize the overall activity of major energy generation pathways between WT and PD multi-cell models, we estimated the FluxSum of key metabolites of glycolysis, TCA, OXPHOS and fatty acid oxidation (FAO) (Fig. 4g, Fig. S3c). FluxSum metabolic estimates were calculated by summing up the incoming (producing) fluxes per metabolite. It gives an estimate of the metabolic activity per metabolite or per pathway when summing up key metabolites of the respective pathway (Supplementary Table S5). The analysis indicated higher glycolysis flux in PD multi-cell models, consistent with increased lactate and by-product formation rate, most likely representing astrocyte metabolism. However, the FluxSum for TCA and OXPHOS was reduced in PD organoid models for both time points compared to the WT case. Moreover, FAO showed an overall increased FluxSum in PD models and might play an important role as a compensatory mechanism and alternative acetyl-CoA source for energy generation in the PD-MIRO1.

### Multi-cell population modeling predicts differential activities of core metabolic pathways in WT vs PD conditions

Next, we looked in more detail into each of the core pathways to further identify weak points of PD metabolism at the single-cell level. In glycolysis, despite the predicted higher FluxSum of all metabolites in PD models (Figs. 4g and 5a, Fig. S3d), a reduced glucose uptake (glc_D[c]) was indicated by the analysis. This could represent a limiting factor in PD organoids’ glycolytic ability over time and is consistent with the significantly lower expression of *HK2* observed in PD dopaminergic neurons (Fig. 3h). Next, in the TCA and OXPHOS metabolic pathways, we observed pronounced differences in the flux of different metabolites between WT and PD metabolic models (Fig. 5b, c, Fig. S3e-f). These observations are in line with our previous experiments showing that 30 day old MIRO1 p.R272Q midbrain organoids have significantly lower mitochondrial basal respiration and ATP-linked production (assessed by seahorse) as well as reduced abundance of the metabolite pyruvate and co-factors NAD+ and FAD +. The model predictions also indicated increased FAO metabolism in PD models, driven particularly by an increase in cytosolic acetyl CoA (accoa[c]) flux, possibly generated from various alternative substrates to glucose (Fig. 5d, Fig. S3g). When we looked at the single-cell level, we could observe that each cell type behaves metabolically differently. According to the FBA predictions, the FluxSum of TCA and OXPHOS in WT dopaminergic neuron models decreased over time, while increasing in astrocytes, suggesting a well-established metabolic crosstalk between these two cell types (Fig. S4a, b). Additionally, the analysis indicated higher FluxSum in glycolysis and FAO in WT astrocyte models compared to PD, suggesting increased metabolic capabilities of WT astrocytes (Fig. S4b). Contrary to the WT case, the PD dopaminergic neurons at day 60, showed high FluxSum in all key energy generation pathways – glycolysis, TCA, OXPHOS and FAO (Fig. S4a). However, the analysis in astrocytes indicated almost no flux in TCA and OXPHOS, and over time, decreased metabolic flux in glycolysis and FAO (Fig. S4b). Moreover, glucose uptake, indicated by glcD[c], was reduced in the PD astrocyte model of day 60 compared to WT, implying that acetate might be used as a metabolic substrate to replace glucose in PD (Fig. 4d), while WT astrocytes rely on glucose. These results suggest disrupted metabolic interactions between PD dopaminergic neurons and astrocytes. It seems that PD dopaminergic neurons over time lose astrocyte support and need to activate all relevant pathways at the same time to ensure sufficient levels of energy. The analysis also showed that in dopaminergic neurons the TCA flux increase is solely α-Ketoglutarate (akg[m]) dependent, suggesting increased glutamine metabolism, which also can fuel TCA through α-Ketoglutarate. In addition, increased OXPHOS flux was based on mitochondrial NADH (nadh[m]) flux, which can only predict the activity of mitochondrial complex I. This suggests that in PD dopaminergic neurons mitochondrial metabolism is disrupted and cannot be used for complete substrate oxidation to generate ATP. Similarly, we observed strong discrepancies in key metabolic pathways in other cell types between WT and PD models. Particularly, the analysis showed an opposite change of glycolysis and TCA flux in neural progenitors between WT and PD conditions, as well as an increased rate of OXPHOS at both time points and FAO at day 60 in WT models compared to PD. This confirms that already at early stages the metabolic activity is altered in MIRO1 mutant cells (Fig. S5a). Furthermore, there was almost no flux for any of the key energy generation pathways in the PD general neurons at day 60 (Fig. S5b), while the GABAergic neurons seemed to preserve metabolic activity at day 60 but had decreased glycolysis flux (Fig. S6). Altogether, these results suggest that each cell type has a unique metabolic signature, which leads to different coping mechanisms in the disease case.

MIRO1 has an important function in mitochondria homeostasis, with MIRO1 p.R272Q mutation causing mitochondria respiration deficits in midbrain organoids and iPSC-derived dopaminergic neuronal cultures^9,10^. In line with our FluxSum analysis, we were also able to observe predicted OXPHOS and TCA altered function in all cell clusters within the PD multi-cell metabolic models, further supporting MIRO1 key importance in mitochondria bioenergetics. The mitochondrial membrane potential is a key factor in the proton motive force, which plays a central role in aerobic ATP production. Moreover, mitochondrial oxidative phosphorylation is a major generator of ROS, namely at complex I, II and III, which in high levels might drive mitochondria membrane depolarization and impair OXPHOS. Therefore, to confirm the functional status of mitochondria experimentally, we evaluated mitochondrial activity by measuring mitochondrial membrane potential and ROS levels in the whole organoid model. We observed a significantly reduced mitochondrial membrane potential in PD midbrain organoids at all time points from day 20 to day 60 compared to WT midbrain organoids, confirming the overall disrupted ATP synthesis process in PD (Fig. 5e, f). Additionally, we saw significantly increased mitochondrial ROS levels in PD midbrain organoids again consistently across all time points (Fig. 5g, h) as another marker of dysfunctional mitochondrial metabolism. These results confirm the metabolic model predictions, that the MIRO1 p.R272Q mutation has a detrimental effect on mitochondrial metabolic activity, which might be the leading cause of overall metabolic dysregulation resulting in the loss of high energy-demanding dopaminergic neurons.

## Discussion

In this study, we described potential cellular and subcellular causes for MIRO1 p.R272Q mutation-associated dopaminergic neuron selective loss in midbrain organoids. We assessed TH-positive dopaminergic neuron numbers in midbrain organoids and observed their progressive decline over time in the PD-specific organoids. This confirmed our previous observation that the MIRO1 p.R272Q mutation is involved in PD pathology^9^. MIRO1 has a well-established role in mitochondrial transport, function and metabolic activity^5,6^, and is also essential for neurodevelopment, highlighted by the embryonic lethality of MIRO1 knockout in mice^39^. Moreover, central nervous system conditional MIRO1 knockout results in neuronal neurodegeneration, particularly of upper motor neurons, due to mitochondrial dysfunction^39,40^. This suggests a possible link of MIRO1 not only with metabolic processes but also with the neurodevelopment pathway, which seems to be especially important for long and energy-demanding neurons.

At the physiological state, the high energy requirements of dopaminergic neurons^21,22^ are met by ATP generation via TCA and OXPHOS metabolic pathways^41^, using glucose and lactate as substrates. Since lactate is produced by the more glycolytic glial cells^41–43^, the interaction between neurons and glial cells is essential for normal brain metabolism and neural function^44^. In the multi-cell metabolic model analysis presented here, we observed opposite key energy generation pathway activity in WT dopaminergic neurons and astrocytes, demonstrating synchronized metabolic interaction. This pattern was not observed in PD-specific multi-cell metabolic models. FBA of PD models predicted increased acetate usage, which is an important metabolic substrate for astrocytes. In line with our observations, previous reports have shown that certain tissues, including the brain, uptake and use acetate when its concentration in the bloodstream is elevated (fasting conditions), but tend to release it when blood levels are lower (non-fasting conditions)^45^^,^^46^. Additionally, the analysis showed increased lactate and a 3-hydroxybutyrate secretion in PD models at both time points, suggesting high astrocytic metabolic activity and accelerated generation of relevant metabolic substrates for neurons as well as possible metabolic stress. However, at the single-cell level, PD astrocyte metabolic flux in key energy generation pathways was decreased compared to WT. This might indicate that in disease conditions, astrocytes are close to their metabolic limit trying to compensate for dopaminergic neuron metabolic dysfunction in the mutant MIRO1 midbrain organoid model. Interestingly, other cell types, including neural progenitors, general neurons and GABAergic neurons, which also showed metabolic alterations, did not demonstrate synchronized metabolic patterns with astrocytes. This further highlights the dopaminergic neuron vulnerability and the consistent need for metabolic support.

Capitalizing on the fact that the midbrain organoid model is a suitable tool for studying disease-associated processes starting from early neurodevelopment^15,16^, we performed developmental trajectory analysis in the dopaminergic neuronal populations. We observed that the developmental path of dopaminergic neurons is accelerated in MIRO1 p.R272Q mutant organoids. We also identified that the transition between day 30 and day 60 of differentiation is strongly associated with mitochondrial metabolic function. However, in PD dopaminergic neurons, we observed high expression of mitochondrial genes, indicating disrupted mitochondrial biogenesis^47^, which might be the result of metabolic maladaptation over the developmental process caused by the MIRO1 mutation. This would also explain the increased need for metabolic support from glial cells. Experimentally, we confirmed that mitochondrial alterations are observed from day 20 of culture, when dopaminergic neuron loss is not yet observed, suggesting that indeed there are early metabolic adaptations in MIRO1 mutant organoids capable of compensating for the energetic needs up to a tipping point, which results in degeneration of the most energy-sensitive (mature) dopaminergic neurons.

Mitochondrial dysfunction is a well-established pathogenic mechanism of PD^48^. Both rare and common genetic variants have been associated with altered mitochondrial quality and/or impaired mitochondrial respiratory functionality and diminished ability to supply energy^49^. Additionally, we have previously shown that MIRO1 p.R272Q midbrain organoids as well as patient-specific idiopathic PD cellular models have also shown decreased mitochondrial respiratory function accompanied by reduced levels of major metabolic cofactor NAD + ^9,17^.

In addition to altered energy generation, it has been shown that abnormal cholesterol metabolism is involved in PD pathophysiology^50–52^. Metabolic modeling indicated alterations in the mevalonate pathway, which produces several bioactive molecules, including cholesterol. Decreased levels of cholesterol precursor lanosterol have been observed in a PD-mouse model^53^, while PD patient fibroblasts show reduced cholesterol biosynthesis^54^. Brain cholesterol synthesis occurs in situ predominantly by astrocytes since it cannot cross the blood-brain barrier^55^. Cholesterol plays an equally vital role in both the development of the nervous system and in mature neurons, regulating cellular membrane organization, synaptogenesis, and synaptic transmission^51,56^. Interestingly, we observed not only metabolic differences but also altered expression patterns of GFAP and S100B proteins between PD and WT astrocytes, strongly indicating astrocyte dysfunction, possibly affecting their ability to support neurons. Despite being unable to exclude the presence of distinct astrocyte subpopulations in WT vs PD, whose different functions may help explain some of the observed metabolic predicted differences, literature does support astrocyte dysfunction as important in neurodegeneration and PD^57^. Indeed, S100B has an important function in terms of energy metabolism, which might justify the altered neuron-astrocyte crosstalk despite the similar amount of astrocytes between PD and WT midbrain organoids. Curiously, lower S100B protein levels are observed in rats subjected to prenatal stress^58^, while S100B reduced secretion has been associated with energy deficits in attention-deficit hyperactivity disorder^59^. Moreover, a negative correlation between the levels of the PD-related protein α-synuclein and GFAP-positive astrogliosis was observed in PD patient brains, despite their normal amount of astrocytes^60^. During brain development, deficiency in PINK1 leads to a reduction of astrogliogenesis (GFAP-positive cells) in mice^61^. Most notably, Kano and colleagues showed lower proliferation of astrocytes in the human brain (lower GFAP protein levels) as well as in iPSC-derived midbrain organoids (lower GFAP and S100B protein levels) in PD cases caused by mutation in the *PRKN* gene^62^. These evidences support our observations, highlighting the important role of astrocytes in neuron survival.

Generally, astrocytes are considered to be more glycolytic compared to neurons. However, in the multi-cell metabolic models, we observed high OXPHOS and TCA flux, particularly in WT astrocytes compared to the PD case. This observation suggests that a MIRO1 mutation effect on astrocyte mitochondrial metabolism, leading to their partial maturation or functionality, cannot be neglected. In fact, MIRO1 is also expressed in astrocytes^63^. Additionally, the altered functional state of PD-astrocytes might be related to their increased metabolic support to metabolically compromised dopaminergic neurons, exhausting their own functional abilities.

Altogether, the results of our study demonstrate that MIRO1 p.R272Q leads to mitochondrial respiratory dysfunction affecting the dopaminergic neuron developmental path and subsequent metabolic homeostasis, ultimately leading to their loss.

Despite our findings, one needs to consider the study limitations. Only one PD patient line with the MIRO1 p.R272Q mutation was available for the analysis. To improve the statistical power of the results, additional cell lines should be included in future experiments. Future comparisons of such models with other PD patient lines might also allow us to better distinguish between mutation-specific and PD general phenotypes. Furthermore, single-cell metabolic modeling has rather predictive value, and there are currently no methods to experimentally validate predicted metabolic changes at the single-cell level in midbrain organoids. However, metabolic models are generated based on single-cell sequencing data, ensuring model context-specificity based on transcriptomic information and thus, increasing prediction accuracy. In future work, the analysis of the reconstructed metabolic models could be extended by performing flux variability and flux sampling analysis, as well as gene/reaction knockouts as alternative methods to characterize key reactions. Additionally, the contextualization of the transcriptomic data could be accompanied by the manual curation of the cell type models^64^ or by developing cell type-specific biomass functions to further improve the model quality. The most widely used approach for the later (BOFdat^65^) requires multi-omics data sets and ideally cell type-specific essentiality information. As these data sets were not available in our case, we refrained from this approach. Furthermore, based on the high expression similarity between the cell types, we wouldn’t expect many differences in the biomass functions.

Model building is impacted by the high dropout rate of the single-cell transcriptomic data. We have previously addressed this by benchmarking our modeling approach^32^ across 20 different scRNAseq data sets. Specifically, we developed a discretization approach to optimize for compactness, completeness and specificity of the reconstructed metabolic models. Interestingly, the derived optimal parameters were consistent across the different data sets and were accordingly also used in the present study. However, the integration, when possible, of metabolomic data could further improve the model quality. Also, generating models for various time points along the organoid cultures would allow us to consolidate the findings and select key predictions for further experimental validation. Notwithstanding, the significance of single-cell metabolomics in this study lies in its ability to offer insights into the metabolic consequences and potential metabolic interactions at the single-cell level, which we aimed to explore given the experimental evidence of mitochondrial dysfunction in dopaminergic neurons.

## Methods

### Midbrain organoid culture

Two human-derived iPSC lines (Table 1) were used in the current study to derive neuroepithelial stem cells (NESC) as previously described^66^. NESC were cultured in 6-well plates coated with Geltrex (Thermo Fisher Scientific A1413302) in maintenance media: N2B27 base culture media – 50:50 DMEM-F12 (Thermo Fisher Scientific 21331046) and Neurobasal (Thermo Fisher Scientific 10888022), 1% penicillin/streptomycin (Thermo Fisher Scientific 15140122), 1% GlutaMAX (Thermo Fisher Scientific 35050061), 1:100 B27 supplement without vitamin A (Life technologies 12587001) and 1:200 N2 supplement (Thermo Fisher Scientific 17502001) – supplemented with 150 µM ascorbic acid (AA, Sigma A4544), 3 µM CHIR-99021 (Axon Medchem CT99021) and 0.75 µM purmorphamine (PMA, Enzo Life Science ALX-420-045). Midbrain organoids were then generated following our previously described procedure^29,67^. Briefly, 9000 NESC per well were seeded in ultra-low attachment 96 round bottom plates (faCellitate F202003) and, kept in maintenance media for 10 days. Organoids were embedded in 30 µL of Geltrex droplets at day 8, transferred into 24-well ultra-low attachment plates and kept in dynamic conditions (80 rpm) for up to 90 days. From day 10 to 16, organoids were cultured in maturation media (base media further supplemented with 200 µM ascorbic acid, 500 µM dbcAMP (STEMCELL Technologies 100-0244), 10 ng/ml hBDNF (Peprotech 450-02), 10 ng/ml hGDNF (Peprotech 450-10), 1 ng/ml TGF-β3 (Peprotech 100-36E) in the presence of 1 µM purmorphamine. From day 16 onward, organoids were cultured in maturation media. Non-embedded organoids were also used for FACS-based assays. In this case, midbrain organoids were kept in the 96-well ultra-low attachment plates and the culture with maintenance media reduced to 2 days. Mycoplasma contamination was tested monthly using the LookOut Mycoplasma PCR Detection Kit (Sigma MP0035-1KT).

### Immunofluorescence staining

Embedded organoids were fixed overnight at 4 °C in 4% paraformaldehyde and washed 3x with PBS for 15 min. Organoids were embedded in 3% low melting agarose (Biozym Scientific GmbH 840100) and sliced into 70 µm sections using Leica VT1000s vibratome. Blocking and permeabilization of the sections was done in 5% normal goat serum (Thermo Fisher Scientific 10000 C), 0.1% sodium azide and 0.5% Triton X-100 (Carl Roth 3051.3) in PBS at room temperature for 2 h. Sections were incubated for 48 h at 4 °C with primary antibodies—rabbit anti-TH (1:1000, Santa Cruz sc-14007, RRID AB_671397), mouse anti-TUJ1 (1:1000), chicken anti-GFAP (1:1000, Millipore AB5541, RRID AB_177521) and, mouse anti-S100B (1:1000, Sigma S2532, RRID AB_477499)—diluted in 5% goat serum, 0.01% triton X-100 and 0.1% sodium azide in PBS (antibody solution). Sections were washed 3× for 10 min with 0.01% triton X-100 in PBS, followed by incubation for 2 h with secondary antibodies: goat anti-rabbit IgG (H + L) Alexa Fluor™ 488 (1:1000, Thermo Fisher Scientific A-11034, RRID AB_2576217), goat anti-mouse IgM Alexa Fluor™ 568 (Thermo Fisher Scientific A-21043, RRID AB_2535712), goat anti-chicken IgY (H + L) Alexa Fluor™ 647 (Thermo Fisher Scientific A-21449, RRID AB_10374876), goat anit-mouse IgG1 Alexa Fluor™ 488 (Thermo Fisher Scientific A-21121, RRID AB_2535764), goat anit-mouse IgG2a Alexa Fluor™ 568 (Thermo Fisher Scientific A-21134, RRID AB_2535773). Sections were mounted on DBM Teflon® Slides (De Beer Medicals BM-9244) after 3 washes with 0.01% triton X-100 in PBS and 1 in miliQ water and mounted with Fluoromount-G® mounting media (Southern Biotech 0100-01). All incubation steps were done under shaking conditions.

### Organoid sections image acquisition and analysis

Image acquisition and quantification were done using our previously published pipeline^68,69^. Images of the entire organoid sections (x, y and z fields) were acquired with a 20× objective in an automated way by using Yokogawa CV8000 standalone high-content screening confocal microscope and the Cell Voyager and Wako software. Quantification of images was done using adapted in-house developed MATLAB scripts (v.2021a; RRID SCR_001622). Quantification was done in at least one section from at least 2 organoids per cell line from at least 3 independent organoid derivations.

### Flow cytometry analysis

A total of 5 to 10 non-embedded organoids were pulled and dissociated with accutase (Sigma A6964) into single-cell suspension. Briefly, organoids were first incubated for 1 h at 37 °C under an orbital shaker in 300 µl of accutase, then gently triturated with a 1000 µl pipette followed by a 200 µl pipette. Organoids were further incubated for 15 to 25 min and finally dissociated with a 200 µl pipette. Cell suspension was then collected, centrifuged at 400 g for 5 min and washed once with assay media or PBS. Replicates were obtained by pulling different independently culture organoids and at least 3 independent derivations were analyzed per assay for each time point (day 20, 30 and 60) unless stated otherwise.

To assess mitochondrial ROS, samples were stained with Zombie NIR (1:10,000 dilution; Biolegend 423106) and 1 nM MitoSOX Mitochondrial Superoxide Indicators (Invitrogen M36008) diluted in phenol-red free DMEM-F12 for 25 min at 37 °C and 5% CO_2_ conditions. For mitochondrial membrane potential, samples were incubated with Zombie NIR (1: 10,000), 100 nM MitoTracker Green (Invitrogen M7514) and 1 nM TMRM (Invitrogen I34361) in base media for 30 min at 37 °C and 5% CO_2_. Samples were then washed twice with PBS by 400 g centrifugation for 5 min and resuspended in PBS. 10,000 single-cell events were recorded at the BD LSRFortessa (BD Biosciences) using the BD FACSDiva™ Software (BD Biosciences, RRID SCR_001456). The number and intensity of MitoSOX-positive or Mitotracker Green and TMRM double-positive events were analyzed with FlowJo software (v.10.8.1; RRID SCR_008520).

### Single-cell RNA sequencing (scRNAseq)

#### Dissociation of midbrain organoids and single-cell isolation

Embedded midbrain organoids at culture day 30 and 60 were collected, washed with PBS (Gibco 10010-23), and digested on 15 ml conical tube containing 1 ml sCelLive™ Tissue Dissociation Solution (Singleron Biotechnologies 1190062) on a sharker (50 rpm) at 37 °C for 30 min. The obtained cell suspension was filtered (Greiner 542040), centrifuged (350 g for 5 min at 4 °C) and resuspended in 500 µl PBS. Following an Acridine Orange/Propidium Iodide Stain (Logos Biosystems F23001), LUNA-FX7™ Automated Cell Counter (Logos Biosystems) was used to calculate cell number and viability.

#### Preparation and sequencing of the library

The scRNAseq libraries were constructed using GEXSCOPE™ Single Cell RNAseq Library Kit (Singleron Biotechnologies 4161031) according to the manufacturer´s instructions. The mRNA extracted was reverse transcribed into cDNA at 42 °C for 1.5 h, followed by cDNA amplification by PCR. The cDNA was then fragmented and ligated to indexed Illumina adapters. The fragment size distribution of the final amplified library was obtained using the Agilent Fragment Analyzer. The library concentration was calculated using the Qubit 4.0 fluorometer and the libraries were pooled in an equimolar fashion. The single-cell libraries were sequenced on the Illumina NovaSeq 6000 using a 2 × 150-bp (base pair) approach to a final depth of 90 GB per library. The reads were demultiplexed according to the multiplexing index sequencing on Illumina’s BaseCloud platform.

#### Transcriptome data pre-processing

Raw data was generated by pre-processing Fastq data with CeleScope® (v.1.3.0; www.github.com/singleron-RD/CeleScope; Singleron Biotechnologies GmbH, RRID SCR_023553) using default parameters and removing reads with low quality. Sequences were mapped using STAR (https://github.com/alexdobin/STAR) and Ensembl 92 was used to annotate the human reference GRCh38 and genes. Reads were assigned to genes using featureCount (https://subread.sourceforge.net/; RRID SCR_009803) and the cell calling was performed by fitting a negative bimodal distribution and by determining the threshold between empty wells and cell-associated wells, to generate a count matrix file containing the number of Unique Molecular Identifier (UMI) for each gene within each cell.

#### scRNAseq data processing

Downstream analysis was done via Seurat single-cell analysis toolkit (v. 4.2.0; RRID:SCR_016341)^70^ on R v. 4.2.2; RRID:SCR_001905). Cells with less than 300 genes and more than 7000 were excluded to avoid doublets or low-quality. In addition, cells having a mitochondrial gene content above 10 or 15% were considered low-quality cells and excluded. The dataset was integrated using Seurat integration workflow^70^ based on the first 20 PCA components. The integrated dataset was scaled, and applying the Louvain algorithm modularity optimization with a resolution of 0.15, seven distinct cell clusters were identified and visualized using the uniform manifold approximation and projection (UMAP) technique^71^. Marker genes of each cell population were determined by applying the *FindAllMarkers* function of Seurat. Based on identified marker genes for each cellular population, cellular identities were determined using the GeneAnalytics online tool (https://geneanalytics.genecards.org/)^72^ and validated by cell type-specific marker genes described in the literature. Differential gene expression analysis was performed using *DESeq2* test.

#### scRNAseq pseudotime trajectory analysis

Pseudotime trajectory analysis was performed using Monocle (v.3; RRID:SCR_018685)^31^ workflow on an integrated Seurat object or its subset of dopaminergic neurons in R version 4.2.2. For the analysis of the complete Seurat object, including all cell types, neural progenitors were chosen as a starting point for the pseudotime trajectory. For the analysis only of dopaminergic neurons, first, both dopaminergic neuron clusters (dopaminergic neurons and TH^high^ dopaminergic neurons) were subset from the complete object. Then the cluster of dopaminergic neurons was chosen as a starting point of the trajectory, based on the observation that this cluster was closer on the trajectory to neural progenitors, while the TH^high^ dopaminergic neuron cluster was at the end of the pseudotime trajectory of the complete Seurat object. Function graph_test of graph-autocorrelation analysis then was applied to identify the most varying genes over the trajectories between time points of PD or WT samples. The enrichment analysis of genes of interest was performed using MetaCore (v. 2023 Clarivate, RRID SCR_008125).

### Metabolic modeling

Metabolic multi-cell population models were reconstructed with scFASTCORMICS based on the scRNAseq data as described elsewhere^32^. Briefly, scRNAseq data were discretized per cell type and condition with default parameters to obtain sets of cell type and condition-specific core genes. For the different conditions (timepoint and midbrain organoid type), we considered the following cell types: neural progenitors, astrocyte-like glia progenitors, neurons, GABAergic neurons and dopaminergic neurons comprising both the dopaminergic neuron cluster and the TH^high^ dopaminergic neuron cluster. Default parameters are based on best performance across 20 single-cell data sets using a pareto approach optimizing compactness, completeness and specificity of the reconstructed models^32^. Core genes were mapped onto the respective metabolic reactions in the generic input reconstruction Recon3D^24^ which has been multiplicated for each cell type within a specific condition. These expanded input models with mapped core reactions were then completed with fastcore^73^ to obtain a consistent multi-cell population model for each experimental condition. Oligodendrocytes and TH^high^ dopaminergic neurons were excluded from the multi-cell models as these clusters included very few cells and were not common between WT and PD conditions. Information on the available medium components was included during the reconstruction, with the concentrations directly mapped to maximal uptake bounds only after the reconstruction. Maximal oxygen uptake was set to twice of the maximal glucose uptake [https://cobrapy.readthedocs.io/en/latest/media.html]. Maximal uptakes of Deoxythymidine-5’-Triphosphate (Thymidine) and linoleate were set to 1 to allow for biomass production. Jaccard similarity per model was performed as described^74^. Flux Balance Analysis (FBA) was performed under the objective of maximizing the sum of the individual biomass maintenance reactions weighted by the number of cells in each cell type cluster with a minimal maintenance of 10% of the maximal per cell type. This ensures an appropriate distribution of the available medium components for the different cell populations, while guaranteeing a minimal support for each cell type. By minimizing the cardinality (zero norm) of the flux distribution a unique (minimal) solution was obtained. FluxSum metabolic estimates were calculated by summing up the incoming (producing) fluxes per metabolite.

### Statistical analysis

Immunofluorescence staining results were plotted and statistically analyzed using RStudio software (version R 4.3.0, RRID SCR_000432). Shapiro test was used to check for data distribution, followed by the nonparametric Kruskall-Walis test to calculate statistical significance between WT, PD and GC conditions within each time point analyzed. For flow cytometry experiments, GraphPad Prism (version 10.1.2, RRID SCR_002798) was used. After assessing normality using the Shapiro-Wilk test, the nonparametric Mann-Whitney test was used to assess significance within each timepoint between WT and PD conditions. Statistical significance was considered for *p* < 0.05, with * or ^$^ *p* < 0.05, ** or ^##^ *p* < 0.01 and *** or ^$$$^ *p* < 0.001.

### Ethics approval

The use of iPSC, obtained from previous studies, was approved by the local authorities, Comité National d’Ethique de Recherche (CNER No 201411/05 and ERP No 18-082 ivPD) following the European Directive 2010/63/EU.w.

## Supplementary information

Supplementary information

Supplementary File1

## Data Availability

All the data (raw and processed) supporting the conclusions of the manuscript are publicly available at 10.17881/7bn3-xc98. ScRNAseq data is available on the Gene Expression Omnibus (GEO) repository under the accession codes GSE237133 and GSE264097.

## Code availability

Scripts for metabolic model generation and analysis can be found at https://github.com/sysbiolux/MidbrainOrganoid_Miro1_scMetabMod. Scripts for single-cell analysis can be found at https://gitlab.lcsb.uni.lu/dvb/zagare_2024.
