# Supplementary material for: MIRO1 mutation leads to metabolic maladaptation resulting in Parkinson’s disease-associated dopaminergic neuron loss
Source: NPJ Syst Biol Appl. 2025 Apr 17;11:37. doi: 10.1038/s41540-025-00509-x (PMC12006346; doi:10.1038/s41540-025-00509-x)
Supplement: Supplementary file 1 — Supplementary information [file 41540_2025_509_MOESM1_ESM.pdf]

Supplementary Information

MIRO1 mutation leads to metabolic maladaptation resulting in Parkinson's disease-associated dopaminergic neuron loss

Alise Zagare[1*], Thomas Sauter[2*], Kyriaki Barmpa[1], Maria Pacheco[2], Rejko Krüger[3,4], Jens Christian Schwamborn[1*#], Claudia Saraiva[1#]

Affiliations

[1]Developmental and Cellular Biology, Luxembourg Centre for Systems Biomedicine (LCSB), University of Luxembourg, 2, place de l'Université, L-4365 Esch-sur-Alzette, Luxembourg.

[2]Systems Biology group, Luxembourg Centre for Systems Biomedicine (LCSB), University of Luxembourg, 2, place de l'Université, L-4365 Esch-sur-Alzette, Luxembourg.

[3]Translational Neuroscience, Luxembourg Centre for Systems Biomedicine (LCSB), University of Luxembourg, 2, place de l'Université, L-4365 Esch-sur-Alzette, Luxembourg.

[4]Transversal Translational Medicine, Luxembourg Institute of Health (LIH), 1 A-B rue Thomas Edison, L-1445 Strassen, Luxembourg

*Equal contribution

[#]Shared corresponding authorship

jens.schwamborn@uni.lu, c.m.m.saraiva@gmail.com

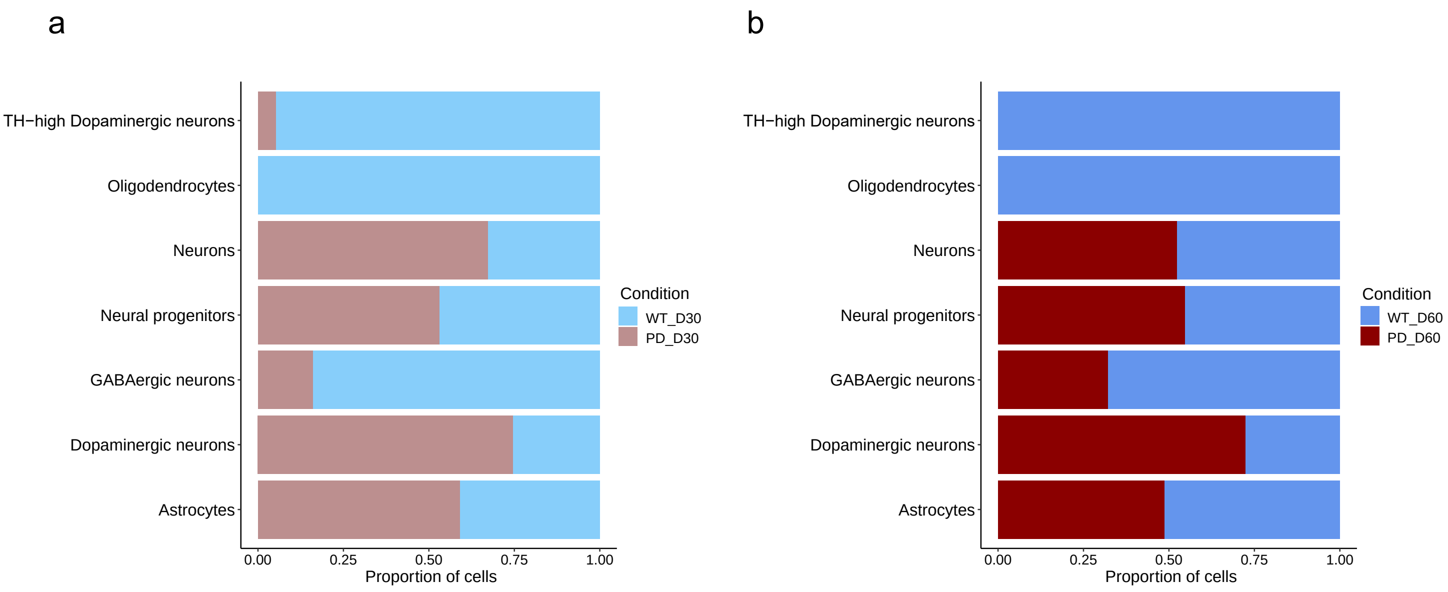

Figure S1. Cell type proportions at different time points. Comparison of the size of each cellular cluster between WT and PD conditions (a) at day 30 (D30) and (b) at day 60 (D60) of organoid culture expressed as a proportion of the total amount of cells belonging to the respective cluster in the single cell RNAseq object.

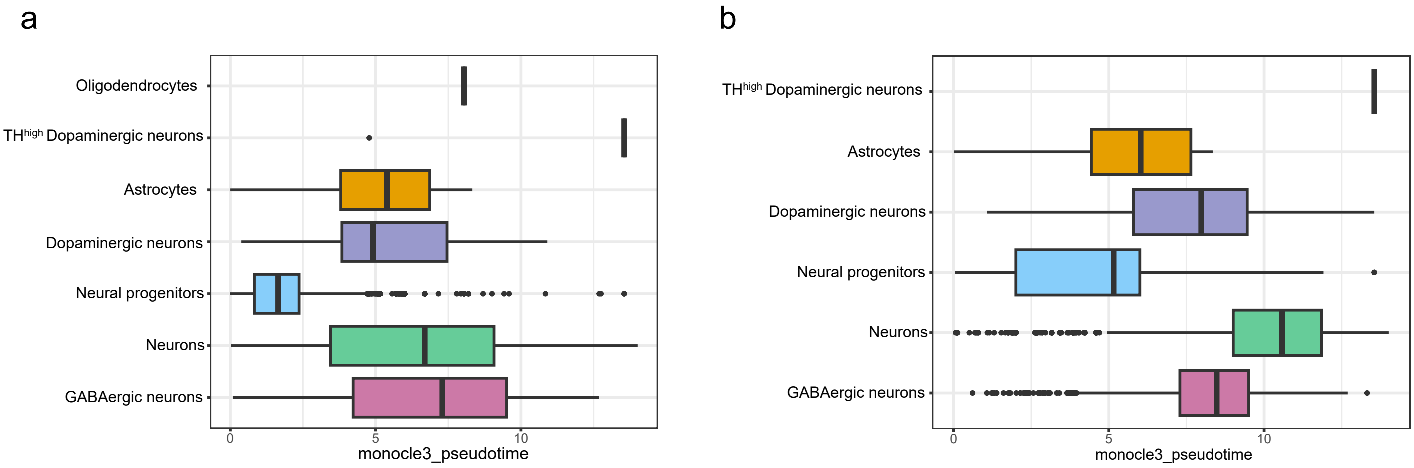

Figure S2. MIRO1 mutant organoids show loss of vulnerable dopaminergic neurons and altered developmental path of cellular populations. (a) Estimated pseudotime for all cell types in WT midbrain organoids (combined day 30 and day 60). (b) Estimated pseudotime for all cell types in PD midbrain organoids (combined day 30 and day 60).

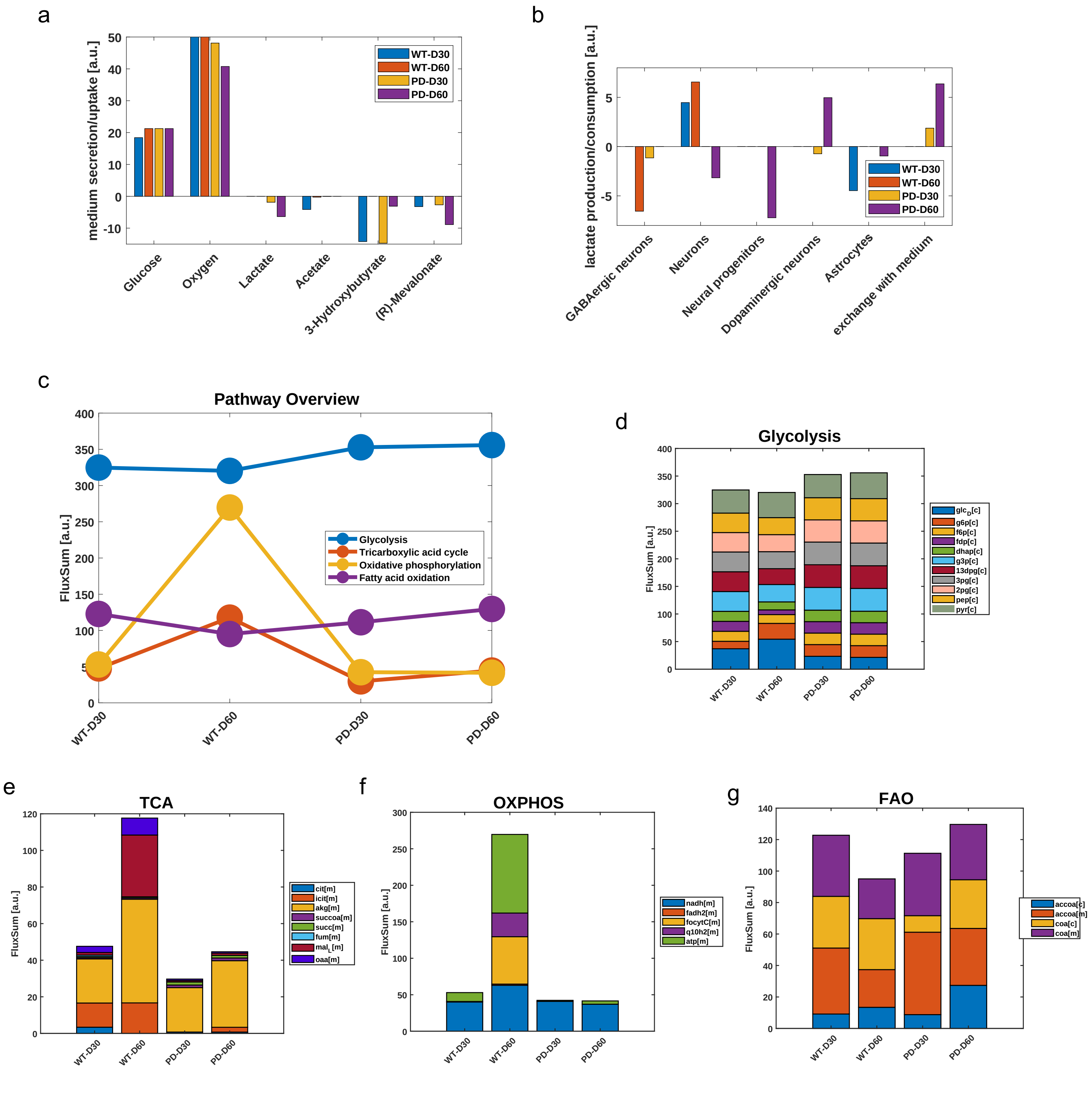

Figure S3. Multi-cell population metabolic modeling predictions are insensitive to maximal oxygen increase. (a) Predicted medium uptake and secretion rates of midbrain organoids for key metabolites. Positive values [a.u.] indicate uptake of the respective metabolite and negative values [a.u.] indicate secretion. (b) Predicted lactate inter-cellular exchange between different cell types. Negative values [a.u.] indicate production of the respective metabolite and positive values [a.u.] indicate uptake (or secretion to the medium). (c) Fluxsum estimates show the metabolic dependence of the midbrain organoid conditions in each energy pathway. Estimates are done by the sum of relevant metabolites (see Table S5) within the respective pathway. (d-g) Metabolic activities are estimated by calculating the FluxSum (in [a.u.]) per metabolite across all cells of the organoid (sum of incoming metabolic fluxes in FBA solution) and are shown as bar plots of key metabolites per metabolic pathway (complete metabolite names corresponding to abbreviations are summarized at Table S5).

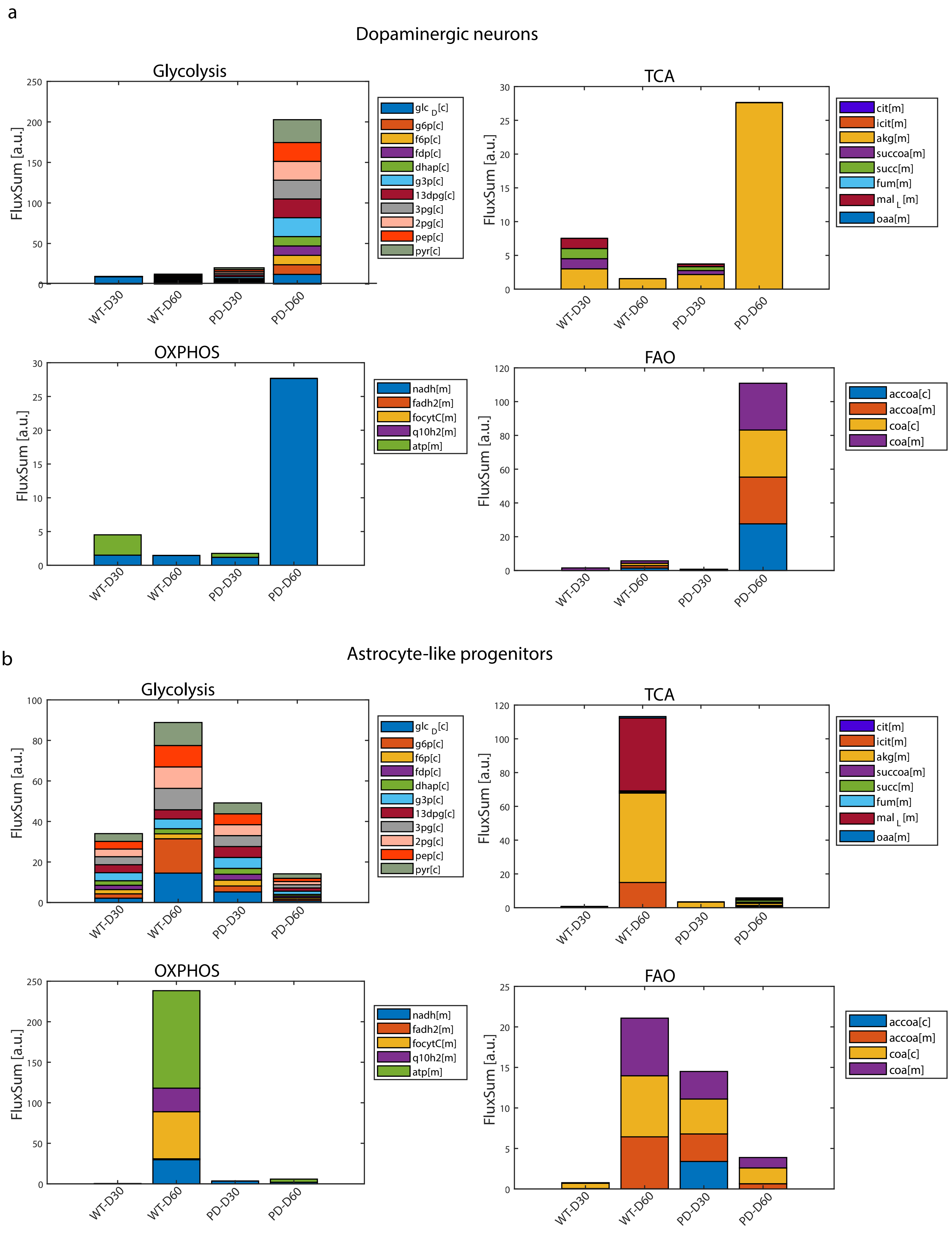

Figure S4. Multi-cell population modeling predicts differential activities of core metabolic pathways in WT vs PD conditions. Estimated FluxSum (in [a.u.]) per metabolite (sum of incoming metabolic fluxes in FBA solution) in (a) dopaminergic neurons and (b) astrocytes and are shown as bar plots of key metabolites per metabolic pathway.

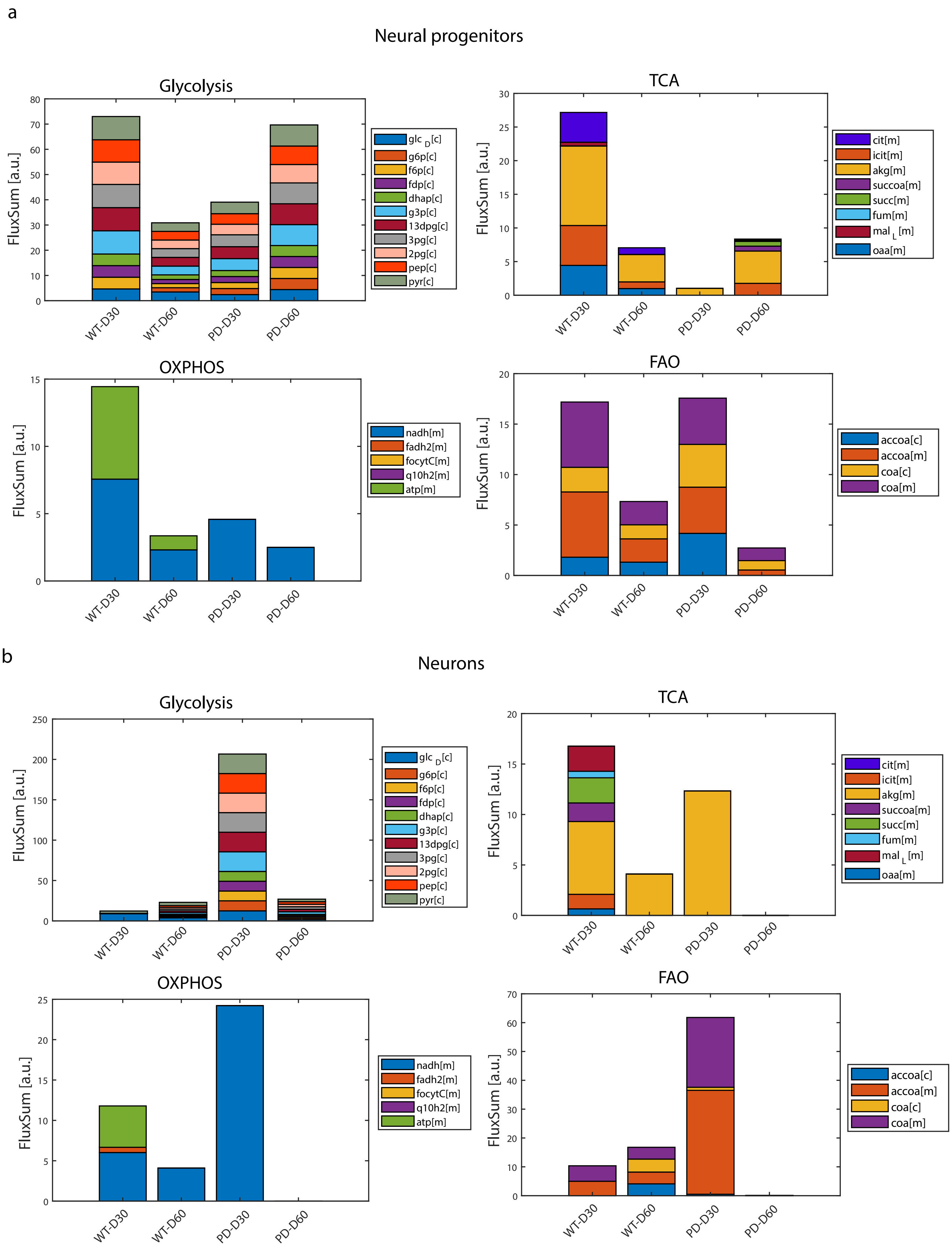

Figure S5. Multi-cell population modeling predicts differential activities of core metabolic pathways in WT vs PD conditions. Estimated FluxSum (in [a.u.]) per metabolite (sum of incoming metabolic fluxes in FBA solution) in (a) neurons and (b) neural progenitor cells and are shown as bar plots of key metabolites per metabolic pathway.

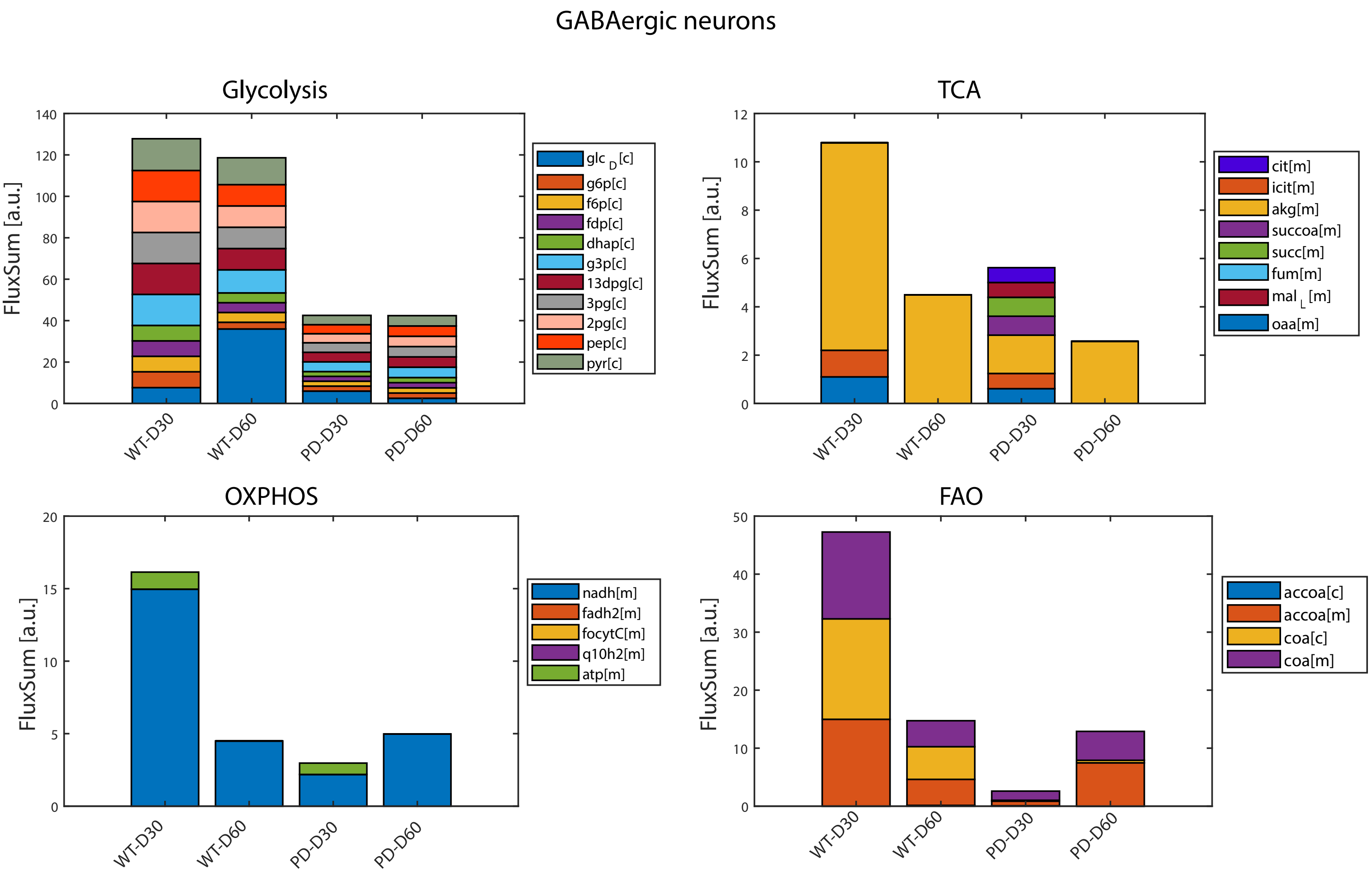

Figure S6. Multi-cell population modeling predicts differential activities of core metabolic pathways in WT vs PD conditions. Estimated FluxSum (in [a.u.]) per metabolite (sum of incoming metabolic fluxes in FBA solution) in GABAergic neurons and are shown as bar plots of key metabolites per metabolic pathway.

Table S1. The top 100 genes with a significant expression change between the two time points - day 30 and day 60 for the WT midbrain organoids.

Gene	p_value	morans_test_statistic	q_value
TAGLN2	0	38.11437	0
TMSB10	0	44.69823	0
SLC5A7	0	58.06673	0
SCG2	0	46.48832	0
GAP43	0	72.92436	0
SST	0	47.13697	0
PHOX2B	0	58.04957	0
AC105389.3	0	39.63936	0
LINC00682	0	47.25301	0
ISL1	0	50.60657	0
SLIT3	0	47.26864	0
NRN1	0	37.86831	0
ARHGAP36	0	38.03508	0
NEFM	0	40.91652	0
NEFL	0	70.73235	0
STMN2	0	47.32075	0
PHOX2A	0	40.38706	0
LAYN	0	41.79922	0
SNCG	0	38.97542	0
CALY	0	38.27564	0
TAC3	0	38.39052	0
RTN1	0	48.37341	0
ANXA2	0	46.8574	0
MAF	0	41.05048	0
GNAS	0	44.89943	0
MT-RNR2	0	37.69207	0
MT-ND1	0	45.7606	0
MT-ND2	0	48.14412	0
MT-CO1	0	63.38316	0
MT-CO2	0	56.38167	0
MT-ATP6	0	46.41531	0
MT-CO3	0	57.01367	0
MT-ND3	0	49.06381	0
MT-ND4L	0	48.60588	0
MT-ND4	0	63.23224	0
MT-ND5	0	57.26671	0
MT-CYB	0	53.05798	0
SNCA	2.01E-305	37.33761	1.10E-302
SH3BGRL3	3.19E-293	36.57806	1.70E-290
CRABP1	5.24E-284	35.99374	2.72E-281

CDKN1A	1.83E-278	35.63762	9.29E-276
MAB21L2	5.27E-276	35.47854	2.61E-273
TLX1	2.57E-272	35.23852	1.24E-269
NHLH1	2.05E-262	34.58598	9.69E-260
UCHL1	1.66E-261	34.52558	7.66E-259
SLC2A1	2.31E-257	34.24833	1.04E-254
SHOX2	1.02E-255	34.13775	4.49E-253
VIM	2.49E-254	34.04397	1.08E-251
MTATP6P1	2.86E-252	33.90447	1.21E-249
SLC17A6	3.90E-248	33.62275	1.62E-245
ID4	2.93E-244	33.35652	1.19E-241
TCF4	1.22E-235	32.75665	4.88E-233
STMN1	2.23E-233	32.59749	8.73E-231
RGMB	1.42E-231	32.46991	5.46E-229
TUBA1B	3.42E-231	32.4428	1.29E-228
H3F3B	3.43E-228	32.22928	1.27E-225
EBF1	2.26E-223	31.88347	8.25E-221
SOX4	1.11E-222	31.83358	3.98E-220
MALAT1	7.53E-218	31.48253	2.65E-215
LHX2	4.90E-210	30.90641	1.70E-207
AC010478.1	9.75E-203	30.35833	3.32E-200
ROBO3	2.13E-196	29.87425	7.12E-194
SLC10A4	3.93E-190	29.3878	1.30E-187
SV2C	5.00E-185	28.98561	1.62E-182
RND3	5.83E-185	28.98029	1.86E-182
DDR1	1.10E-183	28.87885	3.46E-181
DLL1	1.70E-183	28.86381	5.27E-181
MAPT	8.11E-178	28.40777	2.48E-175
S100A6	2.68E-177	28.36569	8.07E-175
TCF12	3.32E-174	28.11378	9.87E-172
CNR1	1.53E-170	27.81254	4.48E-168
TBX2	1.33E-169	27.73474	3.84E-167
FEV	4.36E-168	27.60879	1.24E-165
NEUROD2	4.46E-167	27.52458	1.25E-164
CCK	5.46E-167	27.51723	1.51E-164
SOX11	1.29E-166	27.48592	3.54E-164
LY6H	1.61E-164	27.30997	4.36E-162
LINC01158	3.95E-162	27.10813	1.05E-159
TUBB2A	6.23E-156	26.57721	1.64E-153
UTS2	5.28E-155	26.49678	1.37E-152
RPS27L	6.35E-151	26.14032	1.63E-148

LBX1	6.41E-150	26.05184	1.62E-147
CHL1	3.81E-147	25.80591	9.53E-145
EDIL3	4.99E-139	25.07231	1.23E-136
FTL	1.01E-136	24.85992	2.47E-134
NPPA	1.51E-135	24.75114	3.64E-133
RCAN3	7.14E-135	24.68831	1.71E-132
LAMP5	2.89E-134	24.6317	6.83E-132
BASP1	2.97E-133	24.53704	6.94E-131
ADM	9.42E-133	24.49009	2.17E-130
TFRC	1.03E-128	24.10792	2.36E-126
DLC1	2.50E-128	24.07125	5.65E-126
DCX	3.11E-128	24.06226	6.94E-126
NSG2	1.50E-127	23.99698	3.31E-125
LYPD1	4.24E-126	23.85743	9.27E-124
YWHAH	5.62E-125	23.74901	1.22E-122
MT-RNR1	4.28E-124	23.66355	9.17E-122
RBMS1	8.67E-124	23.63377	1.84E-121
EBF3	1.23E-123	23.61891	2.59E-121
C1orf61	4.80E-123	23.5614	9.97E-121

Table S2. The top 100 genes with a significant expression change between the two time points - day 30 and day 60 for the MIRO1-PD midbrain organoids.

Gene	p_value	morans_test_statistic	q_value
CAMK2N1	0	50.66858	0
STMN1	0	46.66235	0
NHLH1	0	55.7692	0
ATP1B1	0	69.66597	0
CNTN2	0	43.7615	0
LYPD1	0	49.32273	0
RND3	0	61.33213	0
NDNF	0	42.11496	0
HMGCS1	0	39.61499	0
RGMB	0	49.36704	0
PEG10	0	65.40369	0
NEFM	0	37.71724	0
NEFL	0	56.53903	0
DUSP4	0	46.23796	0
BNC2	0	44.04329	0
ROBO3	0	50.96962	0
CRABP1	0	52.96983	0
CBLN1	0	42.36286	0
TOX3	0	51.36249	0
MAF	0	50.36909	0
MAPT	0	43.62036	0
VSTM2L	0	43.73836	0
MAFB	0	43.29908	0
TCF4	1.76E-302	37.15596	1.92E-299
B3GAT2	9.61E-302	37.11021	1.01E-298
TH	1.54E-301	37.09758	1.55E-298
DRGX	1.11E-294	36.66975	1.08E-291
DCC	1.15E-293	36.60602	1.07E-290
LHX2	1.90E-288	36.27647	1.72E-285
MT-CO3	3.48E-287	36.19627	3.04E-284
METRN	1.04E-285	36.10232	8.82E-283
PGM2L1	1.27E-271	35.19315	1.04E-268
SLC35F4	3.41E-269	35.0341	2.70E-266
DCX	1.79E-258	34.3229	1.38E-255
EBF1	4.85E-255	34.092	3.63E-252
AC092376.2	7.88E-255	34.07774	5.74E-252
TMSB4X	2.18E-251	33.84465	1.54E-248
NRN1	1.11E-247	33.59176	7.63E-245
EBF3	2.17E-242	33.22726	1.46E-239
NEUROD2	2.07E-241	33.15942	1.36E-238

TAGLN3	6.80E-241	33.12361	4.34E-238
TUBB2A	3.24E-240	33.07642	2.02E-237
SOX11	6.18E-239	32.98727	3.77E-236
GAP43	1.83E-235	32.74437	1.09E-232
MT-CO1	1.28E-230	32.40213	7.46E-228
MAFA	2.45E-228	32.23968	1.40E-225
CCNI	3.78E-226	32.08323	2.11E-223
ECEL1	2.16E-221	31.74028	1.18E-218
UCHL1	8.68E-218	31.47802	4.65E-215
MT-ND4	2.67E-216	31.36908	1.40E-213
MT-CO2	4.00E-215	31.28279	2.06E-212
ETFB	1.02E-213	31.17912	5.15E-211
MT-CYB	2.64E-209	30.85186	1.31E-206
LGI2	5.36E-209	30.82896	2.60E-206
KITLG	3.70E-194	29.70121	1.77E-191
NFIA	6.24E-190	29.3721	2.92E-187
INSM1	5.58E-187	29.14006	2.57E-184
FTH1	1.13E-186	29.11584	5.11E-184
CRABP2	2.96E-186	29.0828	1.32E-183
CBLN2	5.19E-181	28.66518	2.27E-178
LY6H	3.19E-178	28.44058	1.37E-175
MMD	6.83E-176	28.25146	2.89E-173
TSHZ2	8.07E-175	28.16402	3.36E-172
SOX4	8.86E-171	27.83214	3.63E-168
CDC42	4.43E-169	27.69138	1.79E-166
NPTX2	9.03E-166	27.41523	3.59E-163
NFIB	5.64E-163	27.1797	2.21E-160
ID2	2.85E-162	27.12016	1.10E-159
HES6	1.33E-160	26.97814	5.07E-158
VGF	6.83E-160	26.91762	2.56E-157
NRCAM	2.64E-159	26.86737	9.76E-157
BASP1	4.46E-159	26.84794	1.62E-156
LMO4	9.47E-158	26.73403	3.40E-155
PCDH9	1.42E-154	26.45949	5.03E-152
PCBP4	1.23E-153	26.37794	4.29E-151
C1orf61	9.58E-153	26.30003	3.30E-150
PCDH7	1.16E-152	26.29279	3.95E-150
TMSB10	5.78E-150	26.05578	1.94E-147
EZR	9.35E-149	25.94893	3.10E-146
CHD7	1.59E-144	25.57145	5.19E-142
YWHAH	7.25E-144	25.512	2.35E-141

RBP1	1.97E-143	25.4729	6.22E-141
KCTD12	1.96E-143	25.47304	6.22E-141
IGFBP2	4.81E-143	25.43784	1.50E-140
MT-ATP6	6.25E-143	25.4276	1.93E-140
CHMP2B	7.18E-143	25.42212	2.19E-140
LINC01158	7.64E-143	25.41971	2.30E-140
MEIS2	3.84E-141	25.26535	1.14E-138
DOK6	1.81E-139	25.11261	5.33E-137
CSRP2	4.95E-139	25.07257	1.44E-136
RCOR2	6.42E-138	24.97036	1.85E-135
NGEF	1.03E-137	24.95143	2.94E-135
SNN	6.74E-137	24.87617	1.90E-134
SOX8	3.32E-135	24.71925	9.26E-133
MT-ND5	2.58E-134	24.63624	7.13E-132
CRNDE	3.03E-132	24.44241	8.27E-130
GSTP1	4.81E-132	24.42351	1.30E-129
TSHZ3	1.87E-131	24.36791	5.01E-129
NKAIN4	7.15E-131	24.31297	1.89E-128
CALY	1.75E-130	24.27624	4.58E-128

Table S3. Number of reactions present in each cell-type sub metabolic models.

Nr of reactions	GABAergic neurons	Neurons	Neural progenitors	Dopaminergic neurons	Astrocytes
**WT-D30**	3462	3496	3458	3471	3556
**WT-D60**	3508	3537	3524	3536	3568
**PD-D30**	3441	3510	3492	3471	3572
**PD-D60**	3567	3533	3517	3557	3646

Table S4. Number of metabolites present in each cell-type sub metabolic models.

Nr of metabolites	GABAergic neurons	Neurons	Neural progenitors	Dopaminergic neurons	Astrocytes
**WT-D30**	2464	2465	2442	2450	2463
**WT-D60**	2475	2480	2469	2471	2469
**PD-D30**	2441	2472	2449	2446	2470
**PD-D60**	2496	2479	2461	2489	2488

Table S5. Key metabolites per pathway used for calculating fluxSum of pathways of interest. Abbreviations as used in Recon3D and full names are given for all used metabolites. Letters in brackets indicate the cellular compartment: c, cytoplasm; m, mitochondria.

Pathway	Key metabolite abbreviation	Key metabolite full name
**Glycolysis**	'glc_D[c]'	'D-Glucose'
	'g6p[c]'	'D-Glucose 6-Phosphate'
	'f6p[c]'	'D-Fructose 6-Phosphate'
	'fdp[c]'	'D-Fructose 1,6-Bisphosphate'
	'dhap[c]'	'Dihydroxyacetone Phosphate'
	'g3p[c]'	'Glyceraldehyde 3-Phosphate'
	'13dpg[c]'	'3-Phospho-D-Glyceroyl Phosphate'
	'3pg[c]'	'3-Phospho-D-Glycerate'
	'2pg[c]'	'2-Phospho-D-Glycerate'
	'pep[c]'	'Phosphoenolpyruvate'
	'pyr[c]'	'Pyruvate'
**Tricarboxylic acid cycle (TCA)**	'cit[m]'	'Citrate'
	'icit[m]'	'Isocitric Acid'
	'akg[m]'	'2-Oxoglutarate'
	'succoa[m]'	'Succinyl Coenzyme A'
	'succ[m]'	'Succinate'
	'fum[m]'	'Fumarate'
	'mal_L[m]'	'(S)-Malate'
	'oaa[m]'	'Oxaloacetate'
**Oxidative phosphorylation (OXPHOS)**	nadh[m]'	'Nicotinamide Adenine Dinucleotide - Reduced'
	'fadh2[m]'	'Flavin Adenine Dinucleotide Reduced'
	'focytC[m]'	'Ferrocytochrome C'
	'q10h2[m]'	'Ubiquinol-10'
	'atp[m]'	'Adenosine Triphosphate'
**Fatty acid oxidation (FAO)**	'accoa[c]'	'Acetyl Coenzyme A'
	'accoa[m]'	'Acetyl Coenzyme A'
	'coa[c]'	'Coenzyme A'
	'coa[m]'	'Coenzyme A'
